# Potentially modifiable factors associated with health-related quality of life among people with chronic kidney disease: baseline findings from the National Unified Renal Translational Research Enterprise CKD (NURTuRE-CKD) cohort

**DOI:** 10.1093/ckj/sfae010

**Published:** 2024-01-19

**Authors:** Thomas Phillips, Scott Harris, Olalekan Lee Aiyegbusi, Bethany Lucas, Melissa Benavente, Paul J Roderick, Paul Cockwell, Philip A Kalra, David C Wheeler, Maarten W Taal, Simon D S Fraser

**Affiliations:** School of Primary Care, Population Sciences and Medical Education, Faculty of Medicine, University of Southampton, Southampton, UK; School of Primary Care, Population Sciences and Medical Education, Faculty of Medicine, University of Southampton, Southampton, UK; Centre for Patient-Reported Outcome Research (CPROR), University of Birmingham, Birmingham, UK; Centre for Kidney Research and Innovation, Academic Unit for Translational Medical Sciences, School of Medicine, University of Nottingham, Nottingham, UK; Department of Renal Medicine, Royal Derby Hospital, University Hospitals of Derby and Burton NHS Foundation Trust, Derby, UK; Centre for Kidney Research and Innovation, Academic Unit for Translational Medical Sciences, School of Medicine, University of Nottingham, Nottingham, UK; Department of Renal Medicine, Royal Derby Hospital, University Hospitals of Derby and Burton NHS Foundation Trust, Derby, UK; School of Primary Care, Population Sciences and Medical Education, Faculty of Medicine, University of Southampton, Southampton, UK; Centre for Patient-Reported Outcome Research (CPROR), University of Birmingham, Birmingham, UK; Department of Renal Medicine, Queen Elizabeth Hospital, University Hospitals of Birmingham, Birmingham, UK; Department of Renal Medicine, Salford Royal Hospital, Northern Care Alliance NHS Foundation Trust, Salford, UK; Department of Renal Medicine, University College London, London, UK; Centre for Kidney Research and Innovation, Academic Unit for Translational Medical Sciences, School of Medicine, University of Nottingham, Nottingham, UK; Department of Renal Medicine, Royal Derby Hospital, University Hospitals of Derby and Burton NHS Foundation Trust, Derby, UK; School of Primary Care, Population Sciences and Medical Education, Faculty of Medicine, University of Southampton, Southampton, UK

**Keywords:** chronic kidney disease, health-related quality of life, population health, quality of life

## Abstract

**Background:**

Many non-modifiable factors are associated with poorer health-related quality of life (HRQoL) experienced by people with chronic kidney disease (CKD). We hypothesize that potentially modifiable factors for poor HRQoL can be identified among CKD patients, providing potential targets for intervention.

**Method:**

The National Unified Renal Translational Research Enterprise Chronic Kidney Disease (NURTuRE-CKD) cohort study recruited 2996 participants from nephrology centres with all stages of non-dialysis-dependent CKD. Baseline data collection for sociodemographic, anthropometric, biochemical and clinical information, including Integrated Palliative care Outcome Scale renal, Hospital Anxiety and Depression score (HADS) and the 5-level EuroQol-5D (EQ-5D-5L) as HRQoL measure, took place between 2017 and 2019. EQ-5D-5L dimensions (mobility, self-care, usual activities, pain/discomfort, anxiety/depression) were mapped to an EQ-5D-3L value set to derive index value. Multivariable mixed effects regression models, adjusted for known factors affecting HRQoL with recruitment region as a random effect, were fit to assess potentially modifiable factors associated with index value (linear) and within each dimension (logistic).

**Results:**

Among the 2958/2996 (98.7%) participants with complete EQ-5D data, 2201 (74.4%) reported problems in at least one EQ-5D-5L dimension. Multivariable linear regression identified independent associations between poorer HRQoL (EQ-5D-3L index value) and obesity (body mass index ≥30.0 kg/m^2^, β −0.037, 95% CI −0.058 to −0.016, *P *= .001), HADS depression score ≥8 (β −0.159, −0.182 to −0.137, *P* < .001), anxiety score ≥8 (β −0.090, −0.110 to −0.069, *P* < .001), taking ≥10 medications (β −0.065, −0.085 to −0.046, *P* < .001), sarcopenia (β −0.062, −0.080 to −0.043, *P* < .001) haemoglobin <100 g/L (β −0.047, −0.085 to −0.010, *P *= .012) and pain (β −0.134, −0.152 to −0.117, *P* < .001). Smoking and prescription of prednisolone independently associated with problems in self-care and usual activities respectively. Renin–angiotensin system inhibitor (RASi) prescription associated with fewer problems with mobility and usual activities.

**Conclusion:**

Potentially modifiable factors including obesity, pain, depression, anxiety, anaemia, polypharmacy, smoking, steroid use and sarcopenia associated with poorer HRQoL in this cohort, whilst RASi use was associated with better HRQoL in two dimensions.

## Graphical Abstract

**Graphical Abstract:**
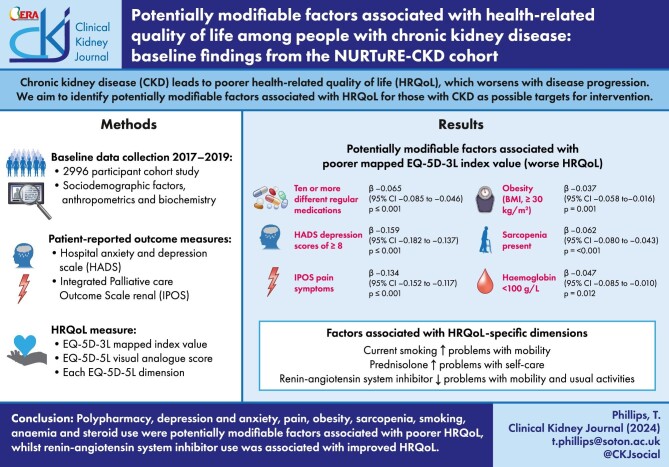

KEY LEARNING POINTS **What was known:**

- Chronic kidney disease (CKD) leads to poorer health-related quality of life (HRQoL), which worsens with disease progression and the need for kidney replacement therapies.
- Sociodemographic, lifestyle and clinical factors are known to influence HRQoL for people with CKD. Poorer HRQoL leads to worse outcomes such as death and CKD progression.

**This study adds:**

- Potentially modifiable factors associated with poorer HRQoL were identified in a non-dialysis dependent cohort. These included obesity, sarcopenia, polypharmacy, anaemia, anxiety, depression, smoking and steroid use, and symptoms such as pain, shortness of breath and weakness.
- Prescription of renin–angiotensin system inhibitor medications associated with improved self-rated mobility and self-care.

**Potential impact:**

1. Many of the factors associated with poorer HRQoL in CKD are potentially modifiable and are possible targets for intervention and further research.
2. In a non-dialysis-dependent cohort of people with CKD managed in secondary care, these factors show that intervention could potentially benefit those with earlier stage CKD.

## INTRODUCTION

Impaired health-related quality of life (HRQoL) is more common among people with chronic kidney disease (CKD) than in the general population [1, 2]. Those with more advanced CKD, especially those requiring kidney replacement therapy (KRT), typically have poorer HRQoL than those with milder disease; this is partly attributable to the treatment burden of KRT [3].

Factors associated with poorer HRQoL in those with non-dialysis-dependent CKD (NDD-CKD) include sociodemographic (lower age, female sex, lack of a partner, lower educational attainment, greater socioeconomic deprivation), lifestyle [higher body mass index (BMI), smoking] and clinical (haemoglobin level, lower clinician-assessed functional status, worse renal function, higher number of comorbidities) factors [3–7]. However, not all of these are modifiable.

Several studies have described associations between poor HRQoL and adverse clinical outcomes [8]. The Renal Impairment In Secondary Care (RIISC) study identified an association between poorer HRQoL and mortality, and higher risk of all-cause and cardiovascular mortality was shown for people with CKD and poor HRQoL in physical domains in the AusDiab cohort [9, 10].

Kidney Disease: Improving Global Outcomes (KDIGO) 2012 guidelines suggested that potentially modifiable factors for CKD progression such as albuminuria, blood pressure, dyslipidaemia, smoking, obesity, physical activity and medications may also impact on HRQoL [11].

A review of qualitative studies and patient-reported outcome measures (PROMs) highlighted that a wide range of symptoms, mood, memory, poor concentration, burden of dietary restrictions and concerns about physical limitations and self-care impact HRQoL for people with CKD, suggesting potential targets for interventions [12].

Given the range of factors influencing HRQoL, and the importance of HRQoL as both an outcome and an indicator of future clinical outcomes, there is a need to identify factors that are potentially modifiable. This includes aspects beyond traditional treatment targets in CKD management. The aim of this study was therefore to identify potentially modifiable factors associated with poorer HRQoL in a large UK cohort of people with CKD managed in secondary care.

## MATERIALS AND METHODS

### Baseline data collection in NURTuRE-CKD

The National Unified Renal Translational Research Enterprise Chronic Kidney Disease (NURTuRE-CKD) cohort began recruitment in 2017. The NURTuRE-CKD HRQoL study was initiated to investigate factors associated with HRQoL outcomes in this cohort.

In total, 2996 participants were enrolled in the NURTuRE-CKD study and baseline data collection took place between 2017 and 2019. The methods are detailed in the NURTuRE-CKD baseline paper [13], and briefly summarized below.

Participants were eligible if aged 18 years or over, had visited a secondary care nephrology clinic at least once and had an estimated glomerular filtration rate (eGFR) as calculated by the CKD Epidemiology Collaboration equation of 15–59 mL/min/1.73 m^2^ or ≥60 mL/min/1.73 m^2^ with a urine albumin to creatinine ratio (uACR) of >30 mg/g. Potential participants also needed to be able to provide informed consent, not be in regular need of any form of KRT, not have received any solid organ transplant and expected to live for >1 year from the time of enrolment. Those on chemotherapy for cancer, with acute kidney injury (AKI) or those who experienced a major cardiovascular event within the previous 3 months were excluded. Recruitment took place in 16 nephrology centres in England, Wales and Scotland (see Table 1).

**Table 1:** Baseline characteristics of participants with complete EQ-5D-5L data.

		Total participants with EQ-5D-5L data	Participants with no problems in any EQ-5D-5L dimension	Participants with problems in any EQ-5D-5L dimension
		*n* (%)^a^	*n* (%)^a^	*n* (%)^a^
Overall number		2958	757 (25.6)	2201 (74.4)
Sex	Male	1729 (58.5)	484 (63.9)	1245 (56.6)
Age at baseline, years (mean ± SD)		62.7 ± 14.7	58.64 ± 15.2	64.1 ± 14.3
Ethnicity	Asian	197 (6.7)	63 (8.3)	134 (6.1)
	Black	89 (3.0)	26 (3.4)	63 (2.9)
	Mixed	31 (1.0)	8 (1.1)	23 (1.0)
	Other	53 (1.8)	20 (2.6)	33 (1.5)
	White	2583 (87.5)	638 (84.5)	1945 (88.5)
Recruitment region	East Midlands	1128 (38.1)	248 (32.8)	880 (40.0)
	London	377 (12.7)	143 (18.9)	234 (10.6)
	North East	196 (6.6)	62 (8.2)	134 (6.1)
	North West	374 (12.6)	84 (11.1)	290 (13.2)
	Scotland	96 (3.2)	37 (4.9)	59 (2.7)
	South East	17 (0.6)	5 (0.7)	12 (0.5)
	Wales	151 (5.1)	39 (5.2)	112 (5.1)
	West Midlands	263 (8.9)	59 (7.8)	204 (9.3)
	Yorkshire and Humber	356 (12.0)	80 (10.6)	276 (12.5)
Renal diagnosis major heading	CKD of uncertain aetiology	952 (32.2)	210 (27.7)	742 (33.8)
	Diabetes mellitus	337 (11.4)	55 (7.3)	282 (12.8)
	Familial/hereditary nephropathies	324 (11.0)	103 (13.6)	221 (10.1)
	Glomerular disease	694 (23.5)	235 (31.0)	459 (20.9)
	Hypertension/renal vascular disease	266 (9.0)	48 (6.3)	218 (9.9)
	Other systemic diseases affecting the kidney	64 (2.2)	15 (2.0)	49 (2.2)
	Tubulointerstitial disease	318 (10.8)	91 (12.0)	227 (10.3)
Had previous KRT		137 (4.6)	21 (2.8)	116 (5.3)
Smoking status	Non-smoker	1479 (50.2)	434 (57.5)	1045 (47.7)
	Ex-smoker	1205 (40.9)	272 (36.0)	933 (42.6)
	Current smoker	263 (8.9)	49 (6.5)	214 (9.8)
Alcohol use		1560 (53.3)	463 (61.7)	1097 (50.3)
Education status	None	829 (29.0)	138 (18.2)	691 (31.4)
	GCSE/NVQ/A-level	1347 (45.5)	334 (44.1)	1013 (46.0)
	Higher education	782 (26.4)	285 (37.6)	497 (22.6)
Employment status	Not in work	348 (11.8)	47 (6.2)	301 (13.7)
	Retired	1582 (53.6)	313 (41.4)	1269 (57.8)
	Working	1020 (34.6)	396 (52.4)	624 (28.4)
IMD quintile	1 = most deprived	641 (21.7)	187 (24.8)	454 (20.7)
	2	612 (20.7)	181 (24.0)	431 (19.6)
	3	549 (18.6)	132 (17.5)	417 (19.0)
	4	543 (18.4)	133 (17.6)	410 (18.7)
	5 = least deprived	607 (20.6)	122 (16.2)	485 (22.1)
EQ-5D-5L dimensions with score 2 or above	Mobility	1465 (49.5)		1465 (66.6)
	Self-care	548 (18.5)		548 (24.9)
	Usual activities	1324 (44.8)		1324 (60.2)
	Pain/discomfort	1773 (59.9)		1773 (80.6)
	Anxiety/depression	1014 (34.3)		1014 (46.1)
EQ-5D-3L mapped index value [median (IQR)]		0.79 (0.36)		0.70 (0.24)
EQ-5D-3L index value less than 0.0		54 (1.8)		54 (2.5)
EQ-5D-5L health rating (visual analogue scale) (mean ± SD)		71.2 ± 20.2	84.93 ± 14.5	66.5 ± 19.8
Number of EQ-5D-5L dimensions scored 2 or above	0 dimensions	757 (25.6)	757 (100)	
	1 dimensions	540 (18.3)		540 (24.5)
	2 dimensions	457 (15.4)		457 (20.8)
	3 dimensions	468 (15.8)		468 (21.3)
	4 dimensions	414 (14.0)		414 (18.8)
	5 dimensions	322 (10.9)		322 (14.6)
SILS score above 2		130 (4.4)	16 (2.1)	114 (5.2)
Number of comorbidities [median (IQR)]		3.0 (3.0)	2.0 (2.0)	3.0 (3.0)
Number of medications 10 or above		948 (32.0)	103 (13.6)	845 (38.4)
Number of regular medications (mean ± SD)		7.8 ± 4.5	5.73 ± 3.5	8.5 ± 4.6
Taking specific medications	RASi	1954 (66.1)	532 (70.3)	1422 (64.6)
	Statins	1716 (58.9)	384 (52.2)	1332 (61.1)
	ESA	186 (6.3)	41 (5.4)	145 (6.6)
	Bicarbonate therapy	347 (11.7)	81 (10.7)	266 (12.1)
	Immunosuppression	308 (10.4)	78 (10.3)	230 (10.4)
	Prednisolone	354 (12.0)	72 (9.5)	282 (12.8)
	Immunosuppression including prednisolone	492 (16.6)	111 (14.7)	381 (17.3)
BMI, kg/m^2^ (mean ± SD)		29.6 ± 6.3	28.09 ± 5.2	30.1 ± 6.5
BMI categories	Underweight (BMI <18.5 kg/m^2^)	32 (1.1)	8 (1.1)	24 (1.1)
	Normal weight (BMI 18.5–24.9 kg/m^2^)	642 (22.3)	202 (27.1)	440 (20.6)
	Overweight (BMI 25.0–29.9 kg/m^2^)	1016 (35.3)	300 (40.3)	716 (33.5)
	Obese (BMI ≥30.0 kg/m^2^)	1190 (41.3)	235 (31.5)	955 (44.7)
KPS [median (IQR)]		90.0 (20.0)	100.0 (10.0)	90.0 (30.0)
MAP, mmHg (mean ± SD)		99.7 ± 12.8	100.73 ± 11.8	99.3 ± 13.1
Sarcopenia present		889 (30.1)	99 (13.1)	790 (35.9)
Admitted to hospital in the last year		885 (29.9)	156 (20.6)	729 (33.1)
eGFR (CKD-EPI formula) central laboratory, mL/min/1.73 m^2^ (mean ± SD)		37.4 ± 17.9	40.66 ± 19.2	36.2 ± 17.3
uACR central laboratory, mg/g [median (IQR)]		206.5 (889.25)	239.0 (886.0)	191.0 (890.0)
Nephrotic-range proteinuria (uACR above 2200 mg/g)		275 (9.3)	70 (9.2)	205 (9.3)
Bicarbonate local laboratory (mmol/L) (mean ± SD)		24.6 ± 3.4	24.64 ± 3.0	24.6 ± 3.5
Bicarbonate <20 mmol/L		172 (5.8)	25 (3.3)	147 (6.7)
Haemoglobin local laboratory, g/L (mean ± SD)		126.9 ± 18.1	131.53 ± 17.5	125.3 ± 18.0
Haemoglobin <100 g/L		147 (5.0)	22 (2.9)	125 (5.7)
Albumin serum local laboratory, g/L (mean ± SD)		40.5 ± 5.3	41.0 ± 5.3	40.3 ± 5.2
Phosphate local laboratory, mmol/L (mean ± SD)		1.1 ± 0.2	1.1 ± 0.2	1.1 ± 0.2
Phosphate above 1.5 mmol/L		121 (4.1)	26 (3.4)	95 (4.3)
PTH local laboratory, pmol/L [median (IQR)]		15.4 (48.0)	12.1 (38.7)	16.68 (51.6)
Calcium adjusted local laboratory, mmol/L (mean ± SD)		2.35 ± 0.1	2.38 ± 0.1	2.36 ± 0.1
PTH categories	Low to normal (0–7.1 pmol/L)	626 (25.4)	191 (30.1)	435 (23.8)
	Raised (7.2–15.7 pmol/L)	618 (25.1)	161 (25.4)	457 (25.0)
	High (15.8–56.0 pmol/L)	626 (25.4)	168 (26.5)	458 (25.1)
	Very high (>56.0 pmol/L)	590 (24.0)	115 (18.1)	475 (26.0)
IPOS symptoms present	Pain	1848 (63.8)	168 (22.6)	1680 (78.1)
	Shortness of breath	1622 (56.2)	224 (29.9)	1398 (65.4)
	Weakness or lack of energy	2125 (72.5)	319 (42.3)	1806 (83.0)
	Nausea	711 (24.4)	73 (9.7)	638 (29.5)
	Vomiting	285 (9.7)	24 (3.2)	261 (12.0)
	Poor appetite	814 (27.8)	82 (10.9)	732 (33.7)
	Constipation	813 (27.8)	96 (12.7)	717 (33.1)
	Mouth problems	562 (19.3)	68 (9.0)	494 (22.9)
	Drowsiness	1370 (47.0)	175 (23.2)	1195 (55.3)
	Poor mobility	1393 (47.7)	42 (5.6)	1351 (62.3)
	Itching	1189 (40.9)	205 (27.3)	984 (45.6)
	Difficulty sleeping	1534 (52.4)	267 (35.5)	1267 (58.3)
	Restless legs	1110 (38.0)	150 (19.9)	960 (44.3)
	Anxiety	1186 (40.6)	132 (17.6)	1054 (48.5)
	Depressive	914 (31.3)	62 (8.2)	852 (39.3)
	Changes in skin	881 (30.3)	131 (17.4)	750 (34.9)
	Diarrhoea	545 (18.7)	72 (9.5)	473 (21.9)
HADS anxiety score ≥8		709 (24.0)	54 (7.1)	655 (29.8)
HADS depression score ≥8		563 (19.0)	20 (2.6)	543 (24.7)
6CIT score ≥8		237 (8.1)	26 (3.4)	211 (9.7)

^a^Unless otherwise stated.

For more detail on variables, the full cohort of 2996 participants and missing data, please refer to Materials and methods and Supplementary data. Percentages here exclude missing data unless specified.

Minor discrepancies in fields such as diagnosis/ethnicity may be seen between this table and the Taal *et al.* (2023) baseline paper—this is due to minor differences in classification and due to only including those with complete EQ-5D-5L data in this table.

MAP, mean arterial pressure; CKD-EPI, CKD Epidemiology Collaboration.

The baseline study visit collected sociodemographic data, medical history, blood and urine test results from both local laboratories and centrally analysed samples (Roche, Geneva, Switzerland), anthropometric measures and several PROMs [13].

The Hospital Anxiety and Depression score (HADS) [14] defines problems with anxiety or depression as scores of 8 or above in either domain. The Six-item Cognitive Impairment Test (6CIT) [15] defines problems with cognition as scores of 8 or above. The Integrated Palliative care Outcome Score for renal (IPOS-renal) [16] was used to measure symptom severity. Health literacy was assessed via the Single-Item Literacy Screener (SILS) [17]. This comprises one question, ‘How often do you need to have someone help you when you read instructions, pamphlets, or other written material from your doctor or pharmacy?’, with responses on an ordinal scale from ‘1—Never’ to ‘5—Always’. Potential health literacy issues are defined as a score of ‘3—Sometimes’ or above.

The Index of Multiple Deprivation (IMD) describes the relative socioeconomic deprivation of each small area in the UK based on its income, employment, education, skills and training, health and disability, crime, barriers to housing and services and living environment [18]. This was obtained for all participants residing in England based on their postal address. As IMD in Wales and Scotland is calculated differently, these were adjusted using the method outlined by Abel *et al*. [19] to allow direct comparison.

Sarcopenia was classified by applying the European Working Group on Sarcopenia in Older People 2 (EWGSOP2) criteria [20] to the results of participants’ timed up and go and hand grip strength tests (with sarcopenia present if either result met criteria). Medications including renin–angiotensin system inhibitors (RASi), immunosuppressive therapies, bicarbonate, phosphate binders and erythropoietin-stimulating agents (ESA) are listed in Supplementary data, Table S1. Nephrotic-range proteinuria was defined as uACR of >2200 mg/g. The Karnofsky Performance Score (KPS) is a validated clinician assessment of functional status on a 0–100 scale where 0 is ‘Dead’ and 100 is ‘Normal no complaints; no evidence of disease’ [21].

BMI was divided into well-recognized categories [22] due to its non-linear relationship with clinical outcomes. Parathyroid hormone (PTH) was similarly categorized into quartiles for analysis due to known associations with poor outcomes at both high and low levels. Current guidance on target PTH levels focuses on overall trend and is numerically specific only for those on dialysis [11, 23]. Only three participants had PTH levels below the normal range (<1.3 pmol/L), therefore the lowest category is amalgamated as ‘low to normal’.

Polypharmacy has varying definitions [24, 25]. A threshold of 10 or more medications was used, due to high numbers of regular medications in this cohort.

Potentially modifiable factors were defined as those which may be reasonably changed by people with CKD or healthcare providers either by lifestyle modification or clinical intervention. These were discussed with a patient advisory group to establish the feasibility of modification (e.g. depression was felt to be potentially modifiable, but not with medication alone).

For a full list of analysed variables and their descriptions, please see the Supplementary data, Tables S1 and S2.

### HRQoL measure

The 5-level EuroQol-5D (EQ-5D-5L) is a validated measure of health status that can be standardized to different populations [26]. It consists of two aspects: five dimensions (mobility, self-care, usual activities, pain or discomfort, anxiety and depression), each with five levels rated on an ordinal scale from ‘1—no problems’ to ‘5—extreme problems’, and a visual analogue scale (VAS) health rating from 0–100 (where 0 is ‘the worst health you can imagine’ and 100 is ‘the best health you can imagine’).

Overall HRQoL is interpreted as EQ-5D-5L index value, where the answers for each dimension are mapped to a value set, which is country specific. The National Institute for Health and Care Excellence (NICE) advise that UK index values should be translated to the previous EQ-5D-3L value set using a method described by Hernandez Alava *et al*. [27, 28]. EQ-5D-3L mapped index values represent 1.0 as perfect health (no problems in any dimensions) and 0.0 as an equivalent health state to death, with negative values possible (indicating a health state worse than death).

The main outcome variables of interest were:

- EQ-5D-3L mapped index value (continuous) as an overall measure of HRQoL,
- problems present in any EQ-5D-5L dimension (measured as two or above in any dimension, binary variable),
- the participants’ self-rated health via the VAS measure,
- problems present within each EQ-5D-5L dimension (measured as score of two or above in each dimension, binary variable).

The number of dimensions which scored two of above was also reported and analysed as a continuous outcome in modelling, however this is not directly comparable with current literature and is a surrogate for index value, therefore the results are reported in Supplementary data, Table S3.

### Statistical analysis

Baseline variables were described using mean and standard deviation (SD) or median and interquartile range (IQR) for continuous variables. Missing data were quantified by number and percentage (see Supplementary data, Tables S1 and S2).

The Python (Python Software Foundation, version 3.9; available at www.python.org) packages Pandas, NumPy and statsmodels were used to prepare data and fit unadjusted univariable logistic (logit) and linear (ordinal least squares) regression models. R (R Foundation for Statistical Computing, version 4.2.2, www.R-project.org) was used for multivariable mixed effects linear and logistic regression using the lme4 and lmerTest packages, as well as assessing for collinearity of covariates using variance inflation factor testing via the cars package.

Independent variables were selected for multivariable regression based on clinical knowledge and the use of direct acyclic graphs to assess the interactions of known variables and create a list of confounders to the potentially modifiable factors of interest (see Supplementary data, Fig. S1). The symptoms of weakness or lack of energy and shortness of breath were chosen as covariates in modelling due to their high prevalence in renal disease and the potential for modification with common existing CKD treatments such as erythropoietin. All multivariable mixed effect logistic and linear regression models were adjusted for these factors including age, sex, ethnicity, IMD, eGFR, number of comorbidities, degree of cognitive impairment and education status. Region of recruitment was added as a random effect to both linear and logistic models to account for clustering.

## RESULTS

Most participants (2958/2996, 98.7%) had complete EQ-5D data. Of these participants, the majority had CKD stage G3a–G4 (*n* = 2573, 87.0%) and 1177 (39.8%) had a uACR of >300 mg/g, with 275 (9.3%) in the nephrotic range. Some 2583 (87.3%) were white and 1729 (58.5%) were male. Mean age was 62.7 (SD ± 14.7) years and the mean number of comorbidities was 3.5 (SD ± 2.2), with 2474 (83.6%) participants diagnosed as hypertensive and 907 (30.7%) diagnosed with diabetes. The mean number of different regular medications was 7.8 (SD ± 4.5), with 1954 (66.1%) taking RASi drugs and 354 (12.0%) taking prednisolone. A total of 889 (30.1%) of participants met the definition for being sarcopenic and 1190 (40.2%) were obese. Table 1 shows the cohort baseline characteristics and Table 2 shows a breakdown of participants’ CKD staging by eGFR and uACR.

**Table 2:** CKD stage of participants, organized by eGFR and uACR for cohort.

	eGFR	A1	A2	A3
	mL/min/1.73 m^2^	<30 mg/g	30–300 mg/g	>300 mg/g
G1	≥90	5 (0.2)	10 (0.4)	37 (1.4)
G2	60–90	74 (2.7)	75 (2.8)	91 (3.3)
G3a	45–60	174 (6.4)	146 (5.4)	166 (6.1)
G3b	30–45	231 (8.5)	314 (11.5)	371 (13.6)
G4	15–30	148 (5.4)	336 (12.3)	486 (17.8)
G5	<15	7 (0.3)	13 (0.5)	42 (1.5)
Due to incomplete ACR data 270 (9.0) participants missing				

Data are presented as *n* (%).

**Figure 1:**
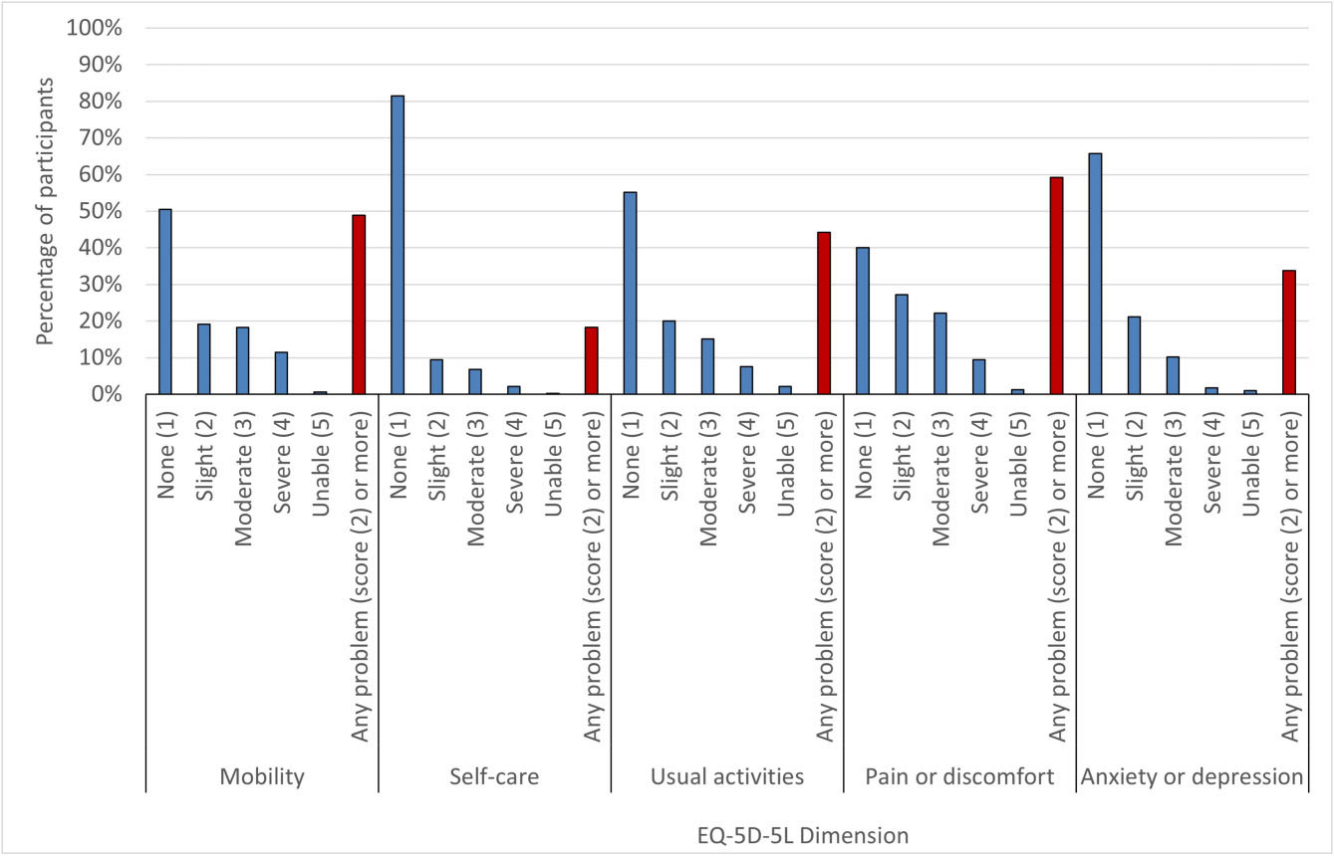
Distribution of the percentage of participants reporting problems within the five dimensions of EQ-5D-5L.

Mean EQ-5D-3L mapped index value was 0.73 (SD ± 0.26) and mean EQ-5D-5L health rating VAS was 71.2/100 (SD ± 20.2). Overall, 2201/2958 (74.4%) reported problems in at least one EQ-5D-5L dimension, with 1465 (49.5%) reporting problems in the mobility dimension, 548 (18.5%) problems with self-care, 1324 (44.8%) problems with usual activities, 1773 (59.9%) problems with pain or discomfort and 1014 (34.3%) problems with anxiety or depression (see Fig. 1). Some 540 (18.3%) participants had problems in one dimension, 457 (15.4%) in two dimensions, 468 (15.8%) in three dimensions, 414 (14.0%) in four dimensions and 322 (10.9%) in all five dimensions.

Univariable linear regression identified associations between EQ-5D-3L mapped index value and multiple variables (see Table 3). Potentially modifiable factors significantly associated with poorer overall HRQoL included worse health literacy (coefficient β −0.165, 95% CI −0.210 to −0.120, *P *<* *.001), being on ≥10 different regular medications (coefficient β −0.199, 95% CI −0.218 to −0.181, *P *<* *.001), statins (coefficient β −0.052, 95% CI −0.071 to −0.033, *P *<* *.001), oral prednisolone (coefficient β −0.035, 95% CI −0.064 to −0.007, *P *= .016), presence of sarcopenia (coefficient β −0.175, 95% CI −0.194 to −0.156, *P *<* *.001), the majority of symptoms in IPOS, significant HADS scores in anxiety (coefficient β −0.222, 95% CI −0.242 to −0.201, *P *<* *.001) and depression (coefficient β −0.305, 95% CI −0.326 to −0.284, *P *<* *.001), haemoglobin <100 g/L (coefficient β −0.120, 95% CI −0.163 to −0.078, *P *<* *.001), very high PTH (>56.0 pmol/L, reference category PTH 0–7.1 pmol/L, coefficient β −0.061, 95% CI −0.090 to −0.032, *P *<* *.001), phosphate (continuous, coefficient β −0.106, 95% CI −0.149 to −0.062, *P *<* *.001) and obesity [BMI ≥30.0 kg/m^2^, reference category normal weight (BMI 18.5–24.9 kg/m^2^), coefficient β −0.102, 95% CI −0.126 to −0.078, *P *<* *.001]. Potentially modifiable factors associated with better HRQoL were taking regular RASi medication (coefficient β 0.035, 95% CI 0.016 to 0.055, *P *<* *.001), higher mean arterial pressure (coefficient β 0.001, 95% CI 0.001 to 0.002, *P *<* *.001) and higher serum albumin (coefficient β 0.003, 95% CI 0.001 to 0.005, *P *= .001).

**Table 3:** Univariable associations with the EQ-5D-3L mapped index value (i.e. overall HRQoL).

	EQ-5D-3L mapped index univariable linear regression				
Risk factor	Coefficient	Lower 95% CI	Upper 95% CI	*P*-value	Association with HRQoL
Sociodemographic					
In work	0.168	0.149	0.186	**<.001**	↑
Has partner	0.048	0.029	0.068	**<.001**	↑
Sex (female)	−0.061	−0.079	−0.042	**<.001**	↓
SILS indicates issue	−0.165	−0.210	−0.120	**<.001**	↓
Medical history					
RASi	0.035	0.016	0.055	**<.001**	↑
Immunosuppression	0.016	−0.015	0.046	.314	*
Bicarbonate therapy	−0.011	−0.040	0.017	.438	*
Phosphate binders	−0.016	−0.057	0.025	.439	*
Prednisolone	−0.035	−0.064	−0.007	**.016**	↓
ESA	−0.072	−0.110	−0.034	**<.001**	↓
Number of comorbidities	−0.044	−0.048	−0.041	**<.001**	↓
Statins	−0.052	−0.071	−0.033	**<.001**	↓
Admitted to hospital in the last year	−0.073	−0.093	−0.053	**<.001**	↓
6CIT score ≥8	−0.118	−0.152	−0.084	**<.001**	↓
Sarcopenia present	−0.175	−0.194	−0.156	**<.001**	↓
Regularly takes over the counter analgesia (paracetamol, co-codamol or ibuprofen)	−0.194	−0.213	−0.176	**<.001**	↓
On ≥10 regular medications	−0.199	−0.218	−0.181	**<.001**	↓
Symptom measures					
IPOS diarrhoea rating	−0.076	−0.088	−0.063	**<.001**	↓
IPOS difficulty sleeping rating	−0.084	−0.092	−0.077	**<.001**	↓
IPOS restless legs rating	−0.096	−0.104	−0.087	**<.001**	↓
IPOS drowsiness rating	−0.100	−0.109	−0.092	**<.001**	↓
IPOS constipation rating	−0.106	−0.116	−0.096	**<.001**	↓
IPOS shortness of breath rating	−0.112	−0.120	−0.104	**<.001**	↓
IPOS poor appetite rating	−0.114	−0.124	−0.104	**<.001**	↓
IPOS weakness or lack of energy rating	−0.121	−0.128	−0.114	**<.001**	↓
IPOS pain rating	−0.148	−0.154	−0.142	**<.001**	↓
HADS anxiety score ≥8	−0.222	−0.242	−0.201	**<.001**	↓
HADS depression score ≥8	−0.305	−0.326	−0.284	**<.001**	↓
Clinical measures					
KPS	0.010	0.009	0.010	**<.001**	↑
Albumin serum (g/L)	0.003	0.001	0.005	**.001**	↑
eGFR (mL/min/1.73 m^2^)	0.002	0.001	0.002	**<.001**	↑
Mean arterial pressure (mmHg)	0.001	0.001	0.002	**<.001**	↑
uACR (mg/g)	0.000	0.000	0.000	.474	*
Potassium (mmol/L)	−0.011	−0.029	0.007	.218	*
Nephrotic-range proteinuria	−0.022	−0.054	0.009	.167	*
PTH (pmol/L)—continuous	0.000	0.000	0.000	**<.001**	↓
PTH raised (7.2–15.7 pmol/L)^a^	−0.029	−0.057	0.000	**.048**	↓↓
PTH high (15.8–56.0 pmol/L)^a^	−0.029	−0.057	0.000	**.048**	↓
PTH very high (>56.0 pmol/L)^a^	−0.061	−0.090	−0.032	**<.001**	↓
HbA1c (mmol/mol)	−0.002	−0.003	−0.001	**<.001**	↓
Urea (mmol/L)	−0.005	−0.006	−0.003	**<.001**	↓
BMI (kg/m^2^)—continuous	−0.010	−0.011	−0.008	**<.001**	↓
Underweight (BMI <18.5 kg/m^2^)^b^	0.035	−0.124	−0.055	.449	*
Overweight (BMI 25–29.9 kg/m2)^b^	−0.012	−0.037	−0.013	.358	*
Obese (BMI ≥30 kg/m^2^)^b^	−0.102	−0.126	−0.078	**<.001**	↓
Bicarbonate <20 mmol/L	−0.050	−0.089	−0.010	**.013**	↓
Phosphate (mmol/L)	−0.106	−0.149	−0.062	**<.001**	↓
Haemoglobin <100 g/L	−0.120	−0.163	−0.078	**<.001**	↓

*P*-values in bold indicate significance <.05.

↑Denotes association with improved HRQoL (i.e. higher index value).

↓Denotes association with worse HRQoL (i.e. lower index value).

*Denotes no clear association or lack of significant association.

^a^Reference category of PTH is low to normal (0–7.1 pmol/L).

^b^Reference category of BMI is normal weight (BMI 18.5–24.9 kg/m^2^).

**Figure 2:**
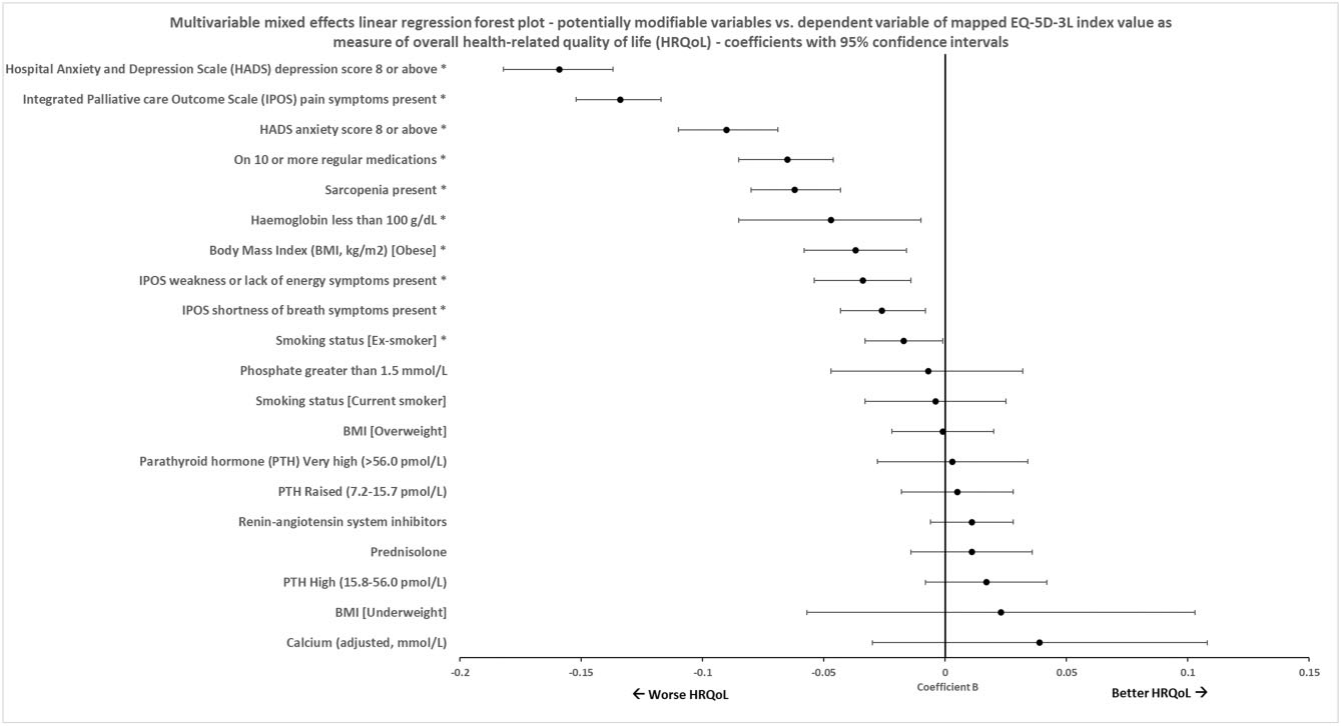
Forest plot of associations with mapped EQ-5D-3L index values in mixed effects multivariable linear regression.

Multivariable mixed effects regression models of EQ-5D-3L mapped index value and health rating VAS (linear) and problems in any EQ-5D-5L dimension (logistic) showed several potentially modifiable variables associated with HRQoL, as shown in Table 4. Figure 2 shows the associations between potentially modifiable variables and index value. Obesity (coefficient β −0.037, 95% CI −0.058 to −0.016, *P *= .001), sarcopenia (coefficient β −0.062, 95% CI −0.080 to −0.043, *P *<* *.001), taking ≥10 regular medications (coefficient β −0.065, 95% CI −0.085 to −0.046, *P *<* *.001), significant HADS scores for depression (coefficient β −0.159, 95% CI −0.182 to −0.137, *P *<* *.001) and anxiety (coefficient β −0.090, 95% CI −0.110 to −0.069, *P *<* *.001), IPOS pain symptoms (coefficient β −0.134, 95% CI −0.152 to −0.117, *P *<* *.001), IPOS shortness of breath symptoms (coefficient β −0.026, 95% CI −0.043 to −0.008, *P *= .005), IPOS weakness or lack of energy symptoms (coefficient β −0.034, 95% CI −0.054 to −0.014, *P *= .001) and haemoglobin of <100 g/L (coefficient β −0.047, 95% CI −0.085 to −0.010, *P *= .012) were independently associated with a worse index value. All these variables also significantly associated with a lower health rating VAS, apart from taking ≥10 regular medications. High PTH (15.8–56.0 pmol/L) was associated with higher health rating VAS [reference category PTH low to normal (0–7.1 pmol/L), coefficient β 3.127, 95% CI 1.113 to 5.141, *P *= .002] and higher adjusted serum calcium (continuous measure) was associated with fewer reported problems in any dimension (odds ratio 0.166, 95% CI 0.049 to 0.560, *P *= .004).

**Table 4:** Potentially modifiable factors in multivariable mixed effects regression models for main HRQoL outcomes.

	EQ-5D-3L mapped index value linear regression			EQ-5D-5L health rating (visual analogue scale) linear regression			EQ-5D-5L problems in any dimension logistic regression			
Risk factors	Coefficient B	95% CI	*P*-values	Coefficient B	95% CI	*P*-values	Odds ratio	95% CI	*P*-values	*n* (%) in cohort with complete EQ-5D-5L data
BMI (kg/m^2^)										
Underweight	0.023	−0.057 to 0.103	.570	−4.301	−11.595 to 2.993	.248	1.171	0.290 to 4.723	.825	32 (1.1)
Overweight	−0.001	−0.022 to 0.020	.916	−0.259	−2.150 to 1.632	.788	0.976	0.703 to 1.357	.887	1016 (35.3)
Obese	−0.037	−0.058 to −0.016	**.001**	−2.747	−4.635 to −0.858	**.004**	1.202	0.856 to 1.687	.288	1190 (41.3)
Smoking status										
Ex-smoker	−0.017	−0.033 to −0.001	**.043**	−0.181	−1.680 to 1.318	.813	1.124	0.857 to 1.475	.397	1205 (40.9)
Current smoker	−0.004	−0.033 to 0.025	.786	0.832	−1.803 to 3.466	.536	1.135	0.666 to 1.934	.641	263 (8.9)
Sarcopenia present	−0.062	−0.080 to −0.043	**<.001**	−4.083	−5.774 to −2.392	**<.001**	1.859	1.322 to 2.615	**<.001**	889 (30.1)
Prednisolone	0.011	−0.014 to 0.036	.404	−1.466	−3.736 to 0.804	.205	0.830	0.540 to 1.275	.395	354 (12.0)
Renin–angiotensin system inhibitors	0.011	−0.006 to 0.028	.200	0.038	−1.486 to 1.563	.961	0.863	0.650 to 1.144	.305	1954 (66.1)
On 10 or more regular medications	−0.065	−0.085 to −0.046	**<.001**	−1.717	−3.484 to 0.049	.057	1.382	0.970 to 1.969	.073	948 (32.0)
Hospital Anxiety and Depression Scale (HADS)										
Depression score 8 or above	−0.159	−0.182 to −0.137	**<.001**	−13.231	−15.296 to −11.165	**<.001**	3.506	1.899 to 6.474	**<.001**	563 (19.0)
Anxiety score 8 or above	−0.090	−0.110 to −0.069	**<.001**	−4.965	−6.842 to −3.087	**<.001**	3.022	1.981 to 4.611	**<.001**	709 (24.0)
Integrated Palliative care Outcome Scale (IPOS)										
Pain symptoms present	−0.134	−0.152 to −0.117	**<.001**	−4.004	−5.602 to −2.406	**<.001**	7.001	5.380 to 9.112	**<.001**	1848 (63.8)
Shortness of breath symptoms present	−0.026	−0.043 to −0.008	**.005**	−2.953	−4.576 to −1.330	**<.001**	1.639	1.236 to 2.173	**.001**	1622 (56.2)
Weakness or lack of energy symptoms present	−0.034	−0.054 to −0.014	**.001**	−7.477	−9.282 to −5.672	**<.001**	2.516	1.904 to 3.325	**<.001**	2125 (72.5)
Haemoglobin <100 g/L	−0.047	−0.085 to −0.010	**.012**	−3.592	−6.968 to −0.217	**.037**	1.239	0.586 to 2.622	.575	147 (5.0)
Phosphate >1.5 mmol/L	−0.007	−0.047 to 0.032	.716	2.542	−1.041 to 6.125	.164	0.944	0.458 to 1.943	.875	121 (4.1)
PTH										
Raised (7.2–15.7 pmol/L)	0.005	−0.018 to 0.028	.668	1.590	−0.455 to 3.634	.127	0.867	0.600 to 1.254	.448	618 (25.1)
High (15.8–56.0 pmol/L)	0.017	−0.008 to 0.042	.190	3.127	1.113 to 5.141	**.002**	0.746	0.495 to 1.123	.160	626 (25.4)
Very high (>56.0 pmol/L)	0.003	−0.028 to 0.034	.841	−0.661	−2.801 to 1.480	.545	0.900	0.547 to 1.482	.678	590 (24.0)
Calcium (adjusted, mmol/L)	0.039	−0.030 to 0.108	.270	3.326	−2.868 to 9.519	.293	0.166	0.049 to 0.560	**.004**	
R^2^ of model	0.513			0.358			0.57			

*P*-values in bold denote statistical significance of <.05.

BMI reference category = normal weight (BMI 18.5–24.9 kg/m^2^); smoking status reference category = non-smoker; PTH reference category = PTH low to normal (0–7.1 pmol/L).

All models include confounding covariates of age*, ethnicity*, sex*, IMD*, eGFR*, number of comorbidities*, 6CIT score of ≥8* as fixed effects and recruitment region as random effect.

*Denotes *P*-value <.05 in any model above (see Supplementary data for effect sizes).

Table 5 summarizes the associations (and direction of association) between potentially modifiable factors and each dimension of EQ-5D-5L. Significant HADS depression scores, IPOS pain symptoms and IPOS weakness or lack of energy symptoms were associated with a greater likelihood of reporting problems in all dimensions, while significant HADS anxiety scores and taking ≥10 regular medications were associated with a higher likelihood of reporting problems in four of the five dimensions. Obesity, sarcopenia and IPOS shortness of breath symptoms associated with more problems in the mobility, self-care and usual activities dimensions, prednisolone use was associated with more problems in the self-care dimension and current smoking associated with problems in the mobility dimension. RASi use was associated with fewer problems in the mobility and usual activities dimensions, whilst higher PTH (15.8–56.0 pmol/L) was associated with fewer issues in usual activities and anxiety and depression dimensions [compared with PTH low to normal (0–7.1 pmol/L)]. Underweight BMI (<18.5 kg/m^2^) associated with fewer problems in the pain or discomfort dimension [compared with normal weight BMI (18.5–24.9 kg/m^2^)] and a higher adjusted serum calcium associated with fewer problems in the mobility dimension.

**Table 5:** Summary of the statistically significant associations between potentially modifiable independent variables and problems in each EQ-5D-5L dimension in multivariable mixed effects logistic regression models.

Risk factors	Mobility dimension	Self-care dimension	Usual activities dimension	Pain/discomfort dimension	Anxiety/depression dimension
HADS depression score ≥8	✓ ↓	✓ ↓	✓ ↓	✓ ↓	✓ ↓
IPOS pain symptoms present	✓ ↓	✓ ↓	✓ ↓	✓ ↓	✓ ↓
IPOS weakness or lack of energy symptoms present	✓ ↓	✓ ↓	✓ ↓	✓ ↓	✓ ↓
HADS anxiety score ≥8		✓ ↓	✓ ↓	✓ ↓	✓ ↓
On ≥10 regular medications	✓ ↓	✓ ↓	✓ ↓		✓ ↓
BMI (kg/m^2^) (obese)	✓ ↓	✓ ↓	✓ ↓		
Sarcopenia present	✓ ↓	✓ ↓	✓ ↓		
IPOS shortness of breath symptoms present	✓ ↓	✓ ↓	✓ ↓		
Prednisolone		✓ ↓			
Smoking					
Smoking status (current smoker)	✓ ↓				
RASis	✓ ↑		✓ ↑		
PTH high (15.8–56.0 pmol/L)			✓ ↑		✓ ↑
BMI (underweight)				✓ ↑	
Calcium (adjusted, mmol/L)	✓ ↑				

^✓^Denotes significant association (*P*-value <.05). Blank fields denote no significant association with that dimension.

↑Denotes odds ratio <1.0 (i.e. less likely to report problems in that dimension).

↓Denotes odds ratio >1.0 (i.e. more likely to report issues in that dimension).

## DISCUSSION

This cross-sectional study of a large cohort of people with CKD referred to secondary care demonstrated that impaired HRQoL was common, with almost three-quarters of participants reporting problems in at least one EQ-5D-5L dimension. Reporting problems in every dimension of HRQoL was relatively uncommon at 10.9%. As reported in previous studies, those with the non-modifiable factors of older age, female sex, cognitive impairment, greater socioeconomic deprivation, lower educational attainment, lower eGFR and increased comorbidity were more likely to report poorer HRQoL. Additionally, our analyses provided several novel insights regarding potentially modifiable factors associated with HRQoL.

### Lifestyle/functional

Obesity was associated with poorer HRQoL, as found in other studies [4]. A recent review highlighted that interventions aimed at reducing BMI, including diet and exercise, weight loss drugs and bariatric surgery, have all been used effectively for weight loss in people with CKD [29], and interventions targeting weight loss in people without CKD have demonstrated prolonged benefit to HRQoL [30].

Sarcopenia is common in the CKD population and has a multifactorial aetiology [31]. Sarcopenia is a significant contributor to physical frailty [32]. Potentially effective interventions include regular resistance exercise and a higher protein diet, especially in combination [33]. Increased angiotensin II is associated with muscle atrophy in CKD [34] and RASi drug use decelerates the decline of renal function, which may explain the benefit in the mobility and usual care dimensions associated with RASi drug use in this cohort.

Current smoking status was associated with reported problems in the mobility dimension. A review has shown consistent association between smoking and poorer HRQoL and mobility across studies, and highlighted that HRQoL improves with smoking cessation [35]. The use of e-cigarettes as nicotine replacement for those with CKD has not been extensively reported in human studies, but may contribute to albuminuria [36, 37].

### Symptoms

Overall, 59.9% of participants reported problems in the EQ-5D-5L pain dimension, with 63.8% reporting pain in the IPOS. Prevalence of pain in a review of studies in NDD-CKD stages G3–5 was 60.7% [38]. Pain can be complex to manage in people with CKD due to limitations in the safe use of analgesic agents, as non-steroidal anti-inflammatory drugs are associated with bleeding and acute kidney injury, and opioids have been associated with poor clinical outcomes [39]. A cautious approach, specific to the type of pain, is recommended, with limited evidence at present for non-pharmacological therapies for those with CKD [40]. Other symptoms, including lack of energy and shortness of breath, were associated with problems in several HRQoL dimensions and are observed in CKD with varied pathophysiology [41]. Specific treatments for common symptoms were collated in a recent article on optimizing symptom control in conservative care, although the focus was on those with severe CKD [42].

Depression was associated with problems in every EQ-5D-5L dimension and overall HRQoL, which suggests pervasion of other HRQoL aspects, a relationship that has been described previously [43]. The interaction of CKD and depression have been linked to poor clinical outcomes [44]. The Chronic Kidney Disease Antidepressant Sertraline Trial (CAST) [45] found that treatment with traditional anti-depressants for those with CKD had minimal effect on depressive symptoms or HRQoL in a NDD-CKD population, whilst a trial in those on dialysis showed improvement in depression scores with either cognitive behavioural therapy or sertraline [46]. Further work is needed to assess the benefit of interventions for depression in people with NDD-CKD.

### Drugs

Polypharmacy was common, with 32% of the cohort taking ≥10 different medications. It was associated with worse overall HRQoL and problems in most dimensions, in keeping with previous studies [47], but this may partly relate to the associated increase in comorbidity. Strategies for medication review in CKD polypharmacy have been trialled, although impact on HRQoL has not yet been demonstrated [48].

RASi drugs were prescribed for 66.1% of the cohort and associated with fewer reported problems in the mobility and usual activities dimensions. Potential mechanisms could be the anti-proteinuric effect of these drugs, however albuminuria was not significantly associated with HRQoL in any analysis. Another explanation may be improved blood pressure control, although lower blood pressure failed to demonstrate a significant association. RASi drugs have proven beneficial effects on left ventricular function and symptoms in heart failure, which may help to explain these findings, as well as its potential relation to sarcopenia, as described above.

Prednisolone was associated with worse HRQoL in the self-care dimension, which may relate to the known associated between glucocorticoid use and muscle atrophy.

### Biochemical measures

Blood markers including high phosphate, low bicarbonate and high PTH associated with worse HRQoL in univariable modelling only. Association with higher phosphate and worse HRQoL was described in the European Quality (EQUAL) study with longitudinal data [7]. Bicarbonate replacement has not been shown to improve HRQoL in a trial of people with CKD stages 3 and 4 [49].

Higher PTH has a complex relationship with HRQoL in this analysis, as it also positively associated with improved HRQoL in some dimensions, although not when at very high levels (>56.0 pmol/L). This may be due to those with moderately higher PTH levels being on less medication to control their PTH, or due to lack of either adynamic bone disease or osteodystrophy at moderately raised values. Whilst these findings possibly suggest that permissively higher PTH in CKD may benefit HRQoL, due to contrasting directions of affect, this more likely reflects the heterogeneity of presentation in those with secondary hyperparathyroidism at differing stages of CKD and the authors strongly advise caution in its interpretation. Higher adjusted serum calcium levels also associated with better HRQoL, but as only 228 (8.5%) of participants had a calcium outside the normal range (2.2–2.6 mmol/L) and only 71 participants had a low calcium, this is difficult to translate to a clinical context other than to recommend that a low calcium is inadvisable. The authors advise that calcium should be considered a necessary covariate due to confounding of PTH, but that the practical implications of this finding are limited. Conventional treatments for secondary hyperparathyroidism in CKD have yet to show an impact on quality of life [50].

Haemoglobin of <100 g/L was associated with poorer overall HRQoL, which is consistent with other studies [1, 5], and anaemia treatment with ESAs, hypoxia-inducible factor–prolyl hydroxylase inhibitors and iron have led to improved HRQoL [51, 52]. Recommended treatments to improve CKD-related anaemia to above haemoglobin levels of 100 g/L are already part of current guidance [11], therefore these findings are unlikely to prompt change in secondary care nephrology practice, although they may highlight their importance for the HRQoL of people being conservatively managed or at an earlier stage of CKD than G4/5.

Higher eGFR was associated with a higher health rating VAS in multivariable analyses. In some studies, worse eGFR has similarly been associated with worse HRQoL, as was the need for KRT [2], however other studies have shown no significant association [1, 4].

### Strengths and limitations

Strengths include that the NURTuRE-CKD cohort consists of a large number of secondary care nephrology patients across the UK, with a diverse range of validated measures. The data were mostly complete (see Supplementary data), and the anthropometric tests used to define sarcopenia are relatively novel compared with other CKD cohort studies.

However, some limitations should be considered in interpreting the data. As these are cross-sectional analyses, causality cannot be inferred from these findings. The variables also have complex relationships not always unmasked with checks for collinearity performed at the time of multivariable regression modelling. Prospective analyses with longitudinal data for change in HRQoL over time may provide more clarity and clinical outcomes for analysis such as death and CKD progression. Local blood and urine test results were used for some variables, resulting in some discrepancies due to lack of standardization between laboratories.

HADS and 6CIT demonstrated associations with HRQoL, however it should be noted that they are predominantly screening tools and not inherently diagnostic without clinical confirmation. Anaemia was associated with worse HRQoL, however this was in the absence of data on participants’ iron stores, which was unfortunately not available. Those participants with underweight BMI had fewer issues with the pain dimension, however only 32 (1.1%) of the cohort met this criterion, therefore caution should be used when interpreting this finding.

Some factors associated with overall HRQoL outcomes in multivariable analysis directly relate to dimensions of EQ-5D-5L and therefore their contribution to overall HRQoL index value is more direct (i.e. a dimension concerns depression, HADS depression score has significant associations with the HRQoL index value which is partly derived from this dimension). Whilst we recognize this relationship, the importance of these findings is their significance across multiple dimensions of EQ-5D-5L, not limited to their most obvious counterpart. Therefore, these variables have remained in models.

EQ-5D-5L does not capture every aspect of HRQoL and has a documented ceiling effect, where more participants tend to answer ‘no problem’, especially in the self-care dimension [53]. CKD-specific measures such as the Kidney Disease Quality Of Life instrument (KDQoL-36) take into account symptoms specific to kidney disease [54], but heterogeneity of measures can hamper comparison between studies, and comparison with the general population or other chronic conditions.

### Summary and recommendations

We have focussed on potentially modifiable factors associated with HRQoL for people with CKD to identify targets for intervention. Factors identified included:

• Depression and anxiety
• Sarcopenia
• Polypharmacy
• Pain, shortness of breath and weakness
• Anaemia
• Smoking
• Obesity
• Steroid use
• RASi drug use (improved HRQoL)

The most important factors to consider for potential intervention may be framed by those interventions which would potentially address a range of the factors above and are simple to deliver to a wide group of patients. Emerging digital health tools could provide exercise, educational, mental wellbeing and lifestyle interventions directly to patients to help to address depression and anxiety symptoms, sarcopenia, smoking cessation, obesity and health literacy [55]. The recent Kidney BEAM trial showed an improvement in mental composite HRQoL scores for those randomized to this tool versus control [56] and points to these types of interventions having potential future impact. From a daily clinical perspective, anaemia management and prioritization of RASi drugs are already common practice, but their impact on HRQoL adds yet another spotlight to their importance for CKD patients of all stages.

Further research should focus on developing and testing interventions to improve HRQoL in this vulnerable population.

## Supplementary Material

sfae010_Supplemental_File

## Data Availability

Data will not be made available due to a data sharing agreement between University of Nottingham and University of Southampton.
