# Supplementary material for: Potentially modifiable factors associated with health-related quality of life among people with chronic kidney disease: baseline findings from the National Unified Renal Translational Research Enterprise CKD (NURTuRE-CKD) cohort
Source: Clin Kidney J. 2024 Jan 19;17(2):sfae010. doi: 10.1093/ckj/sfae010 (PMC10836575; doi:10.1093/ckj/sfae010)

# Supplementary materials

## Table S1. Descriptors of categorical variables in entire cohort

|  | | **n** | **%** | **Note** |
| --- | --- | --- | --- | --- |
| **Total cohort** |  | 2996 | 100.0 |  |
| **Sex** | Male | 1753 | 58.5 |  |
|  | Female | 1243 | 41.5 |  |
| **Ethnicity** | Asian | 200 | 6.7 | Includes asian Indian, asian Pakistani, Chinese, other asian background, asian Bangladeshi |
|  | Black | 91 | 3.0 | Includes other black background, black Carribean, black African |
|  | Mixed | 34 | 1.1 | Includes other mixed background, mixed white and black Carribean/African, mixed white and asian |
|  | Other | 53 | 1.8 | Includes other ethnic background, refused/not stated |
|  | White | 2613 | 87.2 | Includes white british, other white background, white irish |
|  | Missing | 5 | 0.2 |  |
| **Recruitment region** | East Midlands | 1160 | 38.7 |  |
|  | London | 378 | 12.6 |  |
|  | North East | 200 | 6.7 |  |
|  | North West | 374 | 12.5 |  |
|  | Scotland | 96 | 3.2 |  |
|  | South East | 17 | 0.6 |  |
|  | Wales | 151 | 5.0 |  |
|  | West Midlands | 263 | 8.8 |  |
|  | Yorkshire and Humber | 357 | 11.9 |  |
| **Renal diagnosis major heading** | CKD of uncertain aetiology | 964 | 32.2 |  |
|  | Diabetes Mellitus | 346 | 11.5 |  |
|  | Familial / hereditary nephropathies | 327 | 10.9 |  |
|  | Glomerular disease | 698 | 23.3 |  |
|  | Hypertension / Renal vascular disease | 268 | 8.9 |  |
|  | Other systemic diseases affecting the kidney | 64 | 2.1 |  |
|  | Tubulointerstitial disease | 326 | 10.9 |  |
|  | Missing | 3 | 0.1 |  |
| **Had renal biopsy** | No | 1993 | 66.5 |  |
|  | Yes | 923 | 30.8 |  |
|  | Missing | 80 | 2.7 |  |
| **Had previous KRT** | No | 2856 | 95.3 |  |
|  | Yes | 140 | 4.7 |  |
| **CKD stage** | G1A1 | 5 | 0.2 |  |
|  | G1A2 | 10 | 0.3 |  |
|  | G1A3 | 37 | 1.2 |  |
|  | G2A1 | 74 | 2.5 |  |
|  | G2A2 | 75 | 2.5 |  |
|  | G2A3 | 91 | 3.0 |  |
|  | G3aA1 | 174 | 5.8 |  |
|  | G3aA2 | 146 | 4.9 |  |
|  | G3aA3 | 166 | 5.5 |  |
|  | G3bA1 | 231 | 7.7 |  |
|  | G3bA2 | 314 | 10.5 |  |
|  | G3bA3 | 371 | 12.4 |  |
|  | G4A1 | 148 | 4.9 |  |
|  | G4A2 | 336 | 11.2 |  |
|  | G4A3 | 486 | 16.2 |  |
|  | G5A1 | 7 | 0.2 |  |
|  | G5A2 | 13 | 0.4 |  |
|  | G5A3 | 42 | 1.4 |  |
|  | Missing | 270 | 9.0 |  |
| **Diabetes** | No | 2015 | 67.3 |  |
|  | Yes | 922 | 30.8 |  |
|  | Missing | 59 | 2.0 |  |
| **Hypertension** | No | 434 | 14.5 |  |
|  | Yes | 2503 | 83.5 |  |
|  | Missing | 59 | 2.0 |  |
| **CABG** | No | 2833 | 94.6 |  |
|  | Yes | 104 | 3.5 |  |
|  | Missing | 59 | 2.0 |  |
| **Myocardial Infarction** | No | 2658 | 88.7 |  |
|  | Yes | 279 | 9.3 |  |
|  | Missing | 59 | 2.0 |  |
| **Stroke** | No | 2823 | 94.2 |  |
|  | Yes | 114 | 3.8 |  |
|  | Missing | 59 | 2.0 |  |
| **Peripheral Vascular disease** | No | 2812 | 93.9 |  |
|  | Yes | 125 | 4.2 |  |
|  | Missing | 59 | 2.0 |  |
| **Abdominal Aortic Aneurysm** | No | 2928 | 97.7 |  |
|  | Yes | 9 | 0.3 |  |
|  | Missing | 59 | 2.0 |  |
| **Amputation** | No | 2932 | 97.9 |  |
|  | Yes | 5 | 0.2 |  |
|  | Missing | 59 | 2.0 |  |
| **Atrial Fibrillation** | No | 2644 | 88.3 |  |
|  | Yes | 293 | 9.8 |  |
|  | Missing | 59 | 2.0 |  |
| **COPD** | No | 2803 | 93.6 |  |
|  | Yes | 193 | 6.4 | Positive if history of COPD, emphysema, or bronchiectasis |
| **HIV** | No | 2988 | 99.7 |  |
|  | Yes | 8 | 0.3 |  |
| **Heart failure** | No | 2841 | 94.8 |  |
|  | Yes | 155 | 5.2 |  |
| **TIA** | No | 2836 | 94.7 |  |
|  | Yes | 160 | 5.3 |  |
| **Cerebrovascular disease** | No | 2738 | 91.4 |  |
|  | Yes | 258 | 8.6 | Positive if history of stroke or TIA |
| **Dementia** | No | 2988 | 99.7 |  |
|  | Yes | 8 | 0.3 |  |
| **Asthma** | No | 2646 | 88.3 |  |
|  | Yes | 350 | 11.7 |  |
| **Diabetic nephropathy** | No | 2650 | 88.5 |  |
|  | Yes | 346 | 11.5 |  |
| **Diabetic retinopathy** | No | 2599 | 86.7 |  |
|  | Yes | 397 | 13.3 |  |
| **Peripheral neuropathy** | No | 2738 | 91.4 |  |
|  | Yes | 258 | 8.6 |  |
| **Foot ulcers** | No | 2857 | 95.4 |  |
|  | Yes | 139 | 4.6 |  |
| **Diabetes end organ damage** | No | 2417 | 80.7 |  |
|  | Yes | 579 | 19.3 | Positive if diabetes and history of retinopathy, neuropathy or nephropathy |
| **Liver disease mild** | No | 2850 | 95.1 |  |
|  | Yes | 146 | 4.9 | Positive if 'liver disease' or 'hepatic disease' in history |
| **Liver disease moderate to severe** | No | 2995 | 100.0 |  |
|  | Yes | 1 | 0.0 | Positive if history of liver disease and portal hypertension |
| **Cancer history** | No | 2552 | 85.2 |  |
|  | Yes | 444 | 14.8 | Positive if any cancer in history |
| **Gastric ulcer** | No | 2899 | 96.8 |  |
|  | Yes | 97 | 3.2 | Positive if gastric, peptic or duodenal ulcer listed in history |
| **Connective tissue disease** | No | 2674 | 89.3 |  |
|  | Yes | 322 | 10.7 | Positive if history of lupus, vasculitis, Sjogren's, connective tissue disease, rheumatoid, polymyalgia or sarcoid |
| **Smoking status** | Current smoker | 263 | 8.8 |  |
|  | Ex-smoker | 1209 | 40.4 |  |
|  | Non-smoker | 1483 | 49.5 |  |
|  | Missing | 41 | 1.4 |  |
| **Number of daily cigarettes** | 0 | 2745 | 91.6 |  |
|  | 1 = Less than 20 per day | 196 | 6.5 |  |
|  | 2 = Between 20 and 40 per day | 49 | 1.6 |  |
|  | 3 = 40 or more per day | 6 | 0.2 |  |
| **Alcohol use** | No | 1370 | 45.7 |  |
|  | Yes | 1567 | 52.3 |  |
|  | Missing | 59 | 2.0 |  |
| **Alcohol excess** | No | 2660 | 88.8 |  |
|  | Yes | 336 | 11.2 | Consumes more than 14 units per week |
| **Alcohol increasing risk drinking** | No | 2685 | 89.6 |  |
|  | Yes | 311 | 10.4 | Defined as between 14 and 35 units per week for women and between 14 and 50 units per week for men |
| **Alcohol higher risk drinking** | No | 2975 | 99.3 |  |
|  | Yes | 21 | 0.7 | Defined as over 35 units per week for women and over 50 units per week for men |
| **Number of medications 5 or above** | No | 771 | 25.7 |  |
|  | Yes | 2225 | 74.3 |  |
| **Number of medications 10 or above** | No | 2038 | 68.0 |  |
|  | Yes | 958 | 32.0 |  |
| **Angiotensin Converting Enzyme inhibitor (ACE-I)** | No | 1754 | 58.5 |  |
|  | Yes | 1198 | 40.0 |  |
|  | Missing | 44 | 1.5 |  |
| **Angiotensin-II receptor blocker (ARB)** | No | 2050 | 68.4 |  |
|  | Yes | 902 | 30.1 |  |
|  | Missing | 44 | 1.5 |  |
| **Renin-angiotensin system inhibitor (RASi)** | No | 1014 | 33.8 |  |
|  | Yes | 1982 | 66.2 | Defined as taking either ACE-I or ARB |
| **Statins** | No | 1212 | 40.5 |  |
|  | Yes | 1740 | 58.1 |  |
|  | Missing | 44 | 1.5 |  |
| **SGLT2 inhibitor** | No | 2973 | 99.2 |  |
|  | Yes | 23 | 0.8 | Defined as taking either canagliflozin, dapagliflozin or empagliflozin |
| **Erythropoetin Stimulating Agent** | No | 2803 | 93.6 |  |
|  | Yes | 193 | 6.4 | Defined as taking any form of epoetin alpha, epoetin beta or darbopoetin |
| **Phosphate binder** | No | 2829 | 94.4 |  |
|  | Yes | 167 | 5.6 | Defined as taking any calcium or non-calcium based phosphate binder |
| **Bicarbonate therapy** | No | 2645 | 88.3 |  |
|  | Yes | 351 | 11.7 | Defined as taking either sodium or potassium bicarbonate |
| **Immunosuppression** | No | 2683 | 89.6 |  |
|  | Yes | 313 | 10.4 | Defined as taking monoclonal antibodies, calcineurin inhibitors, cyclophosphamide or anti-metabolites |
| **Prednisolone** | No | 2636 | 88.0 |  |
|  | Yes | 360 | 12.0 |  |
| **Immunosuppression including prednisolone** | No | 2496 | 83.3 |  |
|  | Yes | 500 | 16.7 | Defined as taking either immunosuppression as defined above and/or prednisolone |
| **Vitamin D** | No | 2417 | 80.7 |  |
|  | Yes | 579 | 19.3 | Defined as taking any form of 25-hydroxy vitamin D containing medication |
| **Family history of end-stage kidney disease** | No | 2628 | 87.7 |  |
|  | Yes | 316 | 10.5 |  |
|  | Missing | 52 | 1.7 |  |
| **Number of relatives with end-stage kidney disease** | 0 | 2657 | 88.7 |  |
|  | 1 | 282 | 9.4 |  |
|  | 2 | 40 | 1.3 |  |
|  | 3 | 17 | 0.6 |  |
| **Education status** | GCSE/NVQ/A-level | 1351 | 45.1 |  |
|  | Higher education | 783 | 26.1 |  |
|  | None | 862 | 28.8 |  |
| **Employment status** | Not in work | 349 | 11.6 |  |
|  | Retired | 1586 | 52.9 |  |
|  | Working | 1022 | 34.1 |  |
|  | Missing | 39 | 1.3 |  |
| **First language not English** | No | 2741 | 91.5 |  |
|  | Yes | 255 | 8.5 |  |
| **Marital status** | Divorced / Dissolved Civil Partnership | 263 | 8.8 |  |
|  | Married / Civil Partner | 1853 | 61.8 |  |
|  | Not Disclosed | 24 | 0.8 |  |
|  | Separated | 44 | 1.5 |  |
|  | Single | 464 | 15.5 |  |
|  | Widowed / Surviving Civil Partner | 314 | 10.5 |  |
|  | Missing | 34 | 1.1 |  |
| **English literacy** | Good fluency | 2825 | 94.3 | Self-rated item |
|  | Moderate fluency | 75 | 2.5 |  |
|  | Unable to speak or read | 9 | 0.3 |  |
|  | Very weak fluency | 19 | 0.6 |  |
|  | Missing | 68 | 2.3 |  |
| **English fluency less than good** | No | 2893 | 96.6 |  |
|  | Yes | 103 | 3.4 |  |
| **Single item literacy screener (SILS)** | 1 | 2738 | 91.4 |  |
|  | 2 | 49 | 1.6 |  |
|  | 3 | 75 | 2.5 |  |
|  | 4 | 24 | 0.8 |  |
|  | 5 | 31 | 1.0 |  |
|  | Missing | 79 | 2.6 |  |
| **SILS above 2** | No | 2866 | 95.7 |  |
|  | Yes | 130 | 4.3 |  |
| **Index of Multiple Deprivation (IMD) quintile** | 1 | 646 | 21.6 | IMD quintile for Scotland and Wales participants adjusted via Abel 2016 method to allow comparison |
|  | 2 | 617 | 20.6 |  |
|  | 3 | 557 | 18.6 |  |
|  | 4 | 550 | 18.4 |  |
|  | 5 | 620 | 20.7 |  |
|  | Missing | 6 | 0.2 |  |
| **Dietary status** | Low Protein | 32 | 1.1 |  |
|  | Meat free | 142 | 4.7 |  |
|  | Normal mixed | 2619 | 87.4 |  |
|  | Other | 161 | 5.4 |  |
|  | Missing | 42 | 1.4 |  |
| **Low potassium diet** | No | 2964 | 98.9 |  |
|  | Yes | 32 | 1.1 |  |
| **Low phosphate diet** | No | 2994 | 99.9 |  |
|  | Yes | 2 | 0.1 |  |
| **BMI categories** | Healthy weight | 1680 | 56.1 |  |
|  | Obese | 1203 | 40.2 |  |
|  | Underweight | 32 | 1.1 |  |
|  | Missing | 81 | 2.7 |  |
| **Obese (BMI >30 kg/m2)** | No | 1795 | 59.9 |  |
|  | Yes | 1201 | 40.1 |  |
| **Underweight (BMI <18.5 kg/m^2^)** | No | 2964 | 98.9 |  |
|  | Yes | 32 | 1.1 |  |
| **Karnofsky score ≤70** | No | 2414 | 80.6 |  |
|  | Yes | 582 | 19.4 |  |
| **Waist hip ratio high** | No | 1553 | 51.8 | High defined as waist hip ratio > 0.85 if female or >1.0 if male |
|  | Yes | 1443 | 48.2 |  |
| **Waist height ratio high** | No | 1470 | 49.1 | High defined as over 0.59 |
|  | Yes | 1526 | 50.9 |  |
| **Waist height ratio low** | No | 2975 | 99.3 | Low defined as under 0.35 |
|  | Yes | 21 | 0.7 |  |
| **Timed up and go longer than 20 seconds** | No | 2807 | 93.7 |  |
|  | Yes | 189 | 6.3 |  |
| **Hand grip strength low** | No | 2165 | 72.3 | Low defined as best hand grip strength <16kg if female or <27kg if male |
|  | Yes | 831 | 27.7 |  |
| **Sarcopenia present** | No | 2090 | 69.8 | Sarcopenia defined as either hand grip strength low or timed up and go delayed |
|  | Yes | 906 | 30.2 |  |
| **Admitted to hospital in the last year** | No | 2094 | 69.9 |  |
|  | Yes | 902 | 30.1 |  |
| **Number of emergency admissions to hospital in the last year** | 0 | 2372 | 79.2 |  |
|  | 1 | 507 | 16.9 |  |
|  | 2 | 66 | 2.2 |  |
|  | 3 | 27 | 0.9 |  |
|  | 4 | 14 | 0.5 |  |
|  | 5 | 2 | 0.1 |  |
|  | 6 | 2 | 0.1 |  |
|  | 7 | 5 | 0.2 |  |
|  | 9 | 1 | 0.0 |  |
| **Number of elective admissions to hospital in the last year** | 0 | 2666 | 89.0 |  |
|  | 1 | 279 | 9.3 |  |
|  | 2 | 34 | 1.1 |  |
|  | 3 | 10 | 0.3 |  |
|  | 4 | 4 | 0.1 |  |
|  | 5 | 1 | 0.0 |  |
|  | 6 | 1 | 0.0 |  |
|  | 7 | 1 | 0.0 |  |
| **Regularly takes over the counter paracetamol** | No | 2286 | 76.3 |  |
|  | Yes | 710 | 23.7 |  |
| **Regularly takes over the counter co-codamol** | No | 2756 | 92.0 |  |
|  | Yes | 240 | 8.0 |  |
| **Regularly takes over the counter ibuprofen** | No | 2938 | 98.1 |  |
|  | Yes | 58 | 1.9 |  |
| **Regularly takes over the counter analgesia (paracetamol, co-codamol or ibuprofen)** | No | 2100 | 70.1 |  |
|  | Yes | 896 | 29.9 |  |
| **Urine dip blood present** | No | 1563 | 52.2 |  |
|  | Yes | 1180 | 39.4 |  |
|  | Missing | 253 | 8.4 |  |
| **Urine dip protein present** | No | 1256 | 41.9 |  |
|  | Yes | 1520 | 50.7 |  |
|  | Missing | 220 | 7.3 |  |
| **Urine dip leucocytes present** | No | 2248 | 75.0 |  |
|  | Yes | 513 | 17.1 |  |
|  | Missing | 235 | 7.8 |  |
| **Urine dip nitrites present** | No | 2623 | 87.6 |  |
|  | Yes | 135 | 4.5 |  |
|  | Missing | 238 | 7.9 |  |
| **Urine dip glucose present** | No | 2420 | 80.8 |  |
|  | Yes | 342 | 11.4 |  |
|  | Missing | 234 | 7.8 |  |
| **Bicarbonate less than 20 mmol/L** | No | 2821 | 94.2 |  |
|  | Yes | 175 | 5.8 |  |
| **Haemoglobin less than 100 g/dL** | No | 2844 | 94.9 |  |
|  | Yes | 152 | 5.1 |  |
| **Haemoglobin less than 120 g/dL** | No | 1984 | 66.2 |  |
|  | Yes | 1012 | 33.8 |  |
| **Phosphate above 1.5 mmol/L** | No | 2872 | 95.9 | Conversion factors for units: mmol/L to mg/dL ¸ 0.3229 |
|  | Yes | 124 | 4.1 |  |
| **PTH categories** | Low to normal (0-7.1pmol/L) | 629 | 21 |  |
|  | Raised (7.2-15.7pmol/L) | 620 | 20.7 |  |
|  | High (15.8-56.0pmol/L) | 630 | 21 |  |
|  | Very high (>56.0pmol/L) | 615 | 20.5 |  |
|  | Missing | 502 | 16.8 |  |
| **Nephrotic range proteinuria** | No | 2717 | 90.7 |  |
|  | Yes | 279 | 9.3 | Defined as central laboratory urine albumin creatinine ratio over 2200 mg/g |
| **Integrated Palliative care Outcome Scale (IPOS) pain rating** | 0 = not at all, no effect | 1048 | 35.0 |  |
|  | 1 = slightly, but not bothered to be rid of it | 647 | 21.6 |  |
|  | 2 = moderately, limits some activity or concentration | 788 | 26.3 |  |
|  | 3 = severely, activities or concentration markedly affected | 354 | 11.8 |  |
|  | 4 = overwhelmingly, unable to think of anything else | 62 | 2.1 |  |
|  | Missing | 97 | 3.2 |  |
| **IPOS shortness of breath rating** | 0 = not at all, no effect | 1266 | 42.3 |  |
|  | 1 = slightly, but not bothered to be rid of it | 683 | 22.8 |  |
|  | 2 = moderately, limits some activity or concentration | 662 | 22.1 |  |
|  | 3 = severely, activities or concentration markedly affected | 244 | 8.1 |  |
|  | 4 = overwhelmingly, unable to think of anything else | 37 | 1.2 |  |
|  | Missing | 104 | 3.5 |  |
| **IPOS weakness or lack of energy rating** | 0 = not at all, no effect | 807 | 26.9 |  |
|  | 1 = slightly, but not bothered to be rid of it | 775 | 25.9 |  |
|  | 2 = moderately, limits some activity or concentration | 896 | 29.9 |  |
|  | 3 = severely, activities or concentration markedly affected | 376 | 12.6 |  |
|  | 4 = overwhelmingly, unable to think of anything else | 83 | 2.8 |  |
|  | Missing | 59 | 2.0 |  |
| **IPOS nausea rating** | 0 = not at all, no effect | 2209 | 73.7 |  |
|  | 1 = slightly, but not bothered to be rid of it | 414 | 13.8 |  |
|  | 2 = moderately, limits some activity or concentration | 199 | 6.6 |  |
|  | 3 = severely, activities or concentration markedly affected | 76 | 2.5 |  |
|  | 4 = overwhelmingly, unable to think of anything else | 24 | 0.8 |  |
|  | Missing | 74 | 2.5 |  |
| **IPOS vomiting rating** | 0 = not at all, no effect | 2645 | 88.3 |  |
|  | 1 = slightly, but not bothered to be rid of it | 166 | 5.5 |  |
|  | 2 = moderately, limits some activity or concentration | 72 | 2.4 |  |
|  | 3 = severely, activities or concentration markedly affected | 32 | 1.1 |  |
|  | 4 = overwhelmingly, unable to think of anything else | 15 | 0.5 |  |
|  | Missing | 66 | 2.2 |  |
| **IPOS poor appetite rating** | 0 = not at all, no effect | 2115 | 70.6 |  |
|  | 1 = slightly, but not bothered to be rid of it | 439 | 14.7 |  |
|  | 2 = moderately, limits some activity or concentration | 252 | 8.4 |  |
|  | 3 = severely, activities or concentration markedly affected | 94 | 3.1 |  |
|  | 4 = overwhelmingly, unable to think of anything else | 30 | 1.0 |  |
|  | Missing | 66 | 2.2 |  |
| **IPOS constipation rating** | 0 = not at all, no effect | 2114 | 70.6 |  |
|  | 1 = slightly, but not bothered to be rid of it | 465 | 15.5 |  |
|  | 2 = moderately, limits some activity or concentration | 221 | 7.4 |  |
|  | 3 = severely, activities or concentration markedly affected | 97 | 3.2 |  |
|  | 4 = overwhelmingly, unable to think of anything else | 30 | 1.0 |  |
|  | Missing | 69 | 2.3 |  |
| **IPOS mouth problems rating** | 0 = not at all, no effect | 2356 | 78.6 |  |
|  | 1 = slightly, but not bothered to be rid of it | 313 | 10.4 |  |
|  | 2 = moderately, limits some activity or concentration | 152 | 5.1 |  |
|  | 3 = severely, activities or concentration markedly affected | 75 | 2.5 |  |
|  | 4 = overwhelmingly, unable to think of anything else | 24 | 0.8 |  |
|  | Missing | 76 | 2.5 |  |
| **IPOS drowsiness rating** | 0 = not at all, no effect | 1545 | 51.6 |  |
|  | 1 = slightly, but not bothered to be rid of it | 689 | 23.0 |  |
|  | 2 = moderately, limits some activity or concentration | 470 | 15.7 |  |
|  | 3 = severely, activities or concentration markedly affected | 168 | 5.6 |  |
|  | 4 = overwhelmingly, unable to think of anything else | 46 | 1.5 |  |
|  | Missing | 78 | 2.6 |  |
| **IPOS poor mobility rating** | 0 = not at all, no effect | 1530 | 51.1 |  |
|  | 1 = slightly, but not bothered to be rid of it | 465 | 15.5 |  |
|  | 2 = moderately, limits some activity or concentration | 474 | 15.8 |  |
|  | 3 = severely, activities or concentration markedly affected | 353 | 11.8 |  |
|  | 4 = overwhelmingly, unable to think of anything else | 105 | 3.5 |  |
|  | Missing | 69 | 2.3 |  |
| **IPOS itching rating** | 0 = not at all, no effect | 1726 | 57.6 |  |
|  | 1 = slightly, but not bothered to be rid of it | 652 | 21.8 |  |
|  | 2 = moderately, limits some activity or concentration | 339 | 11.3 |  |
|  | 3 = severely, activities or concentration markedly affected | 132 | 4.4 |  |
|  | 4 = overwhelmingly, unable to think of anything else | 67 | 2.2 |  |
|  | Missing | 80 | 2.7 |  |
| **IPOS difficulty sleeping rating** | 0 = not at all, no effect | 1396 | 46.6 |  |
|  | 1 = slightly, but not bothered to be rid of it | 660 | 22.0 |  |
|  | 2 = moderately, limits some activity or concentration | 484 | 16.2 |  |
|  | 3 = severely, activities or concentration markedly affected | 279 | 9.3 |  |
|  | 4 = overwhelmingly, unable to think of anything else | 115 | 3.8 |  |
|  | Missing | 62 | 2.1 |  |
| **IPOS restless legs rating** | 0 = not at all, no effect | 1817 | 60.6 |  |
|  | 1 = slightly, but not bothered to be rid of it | 519 | 17.3 |  |
|  | 2 = moderately, limits some activity or concentration | 358 | 11.9 |  |
|  | 3 = severely, activities or concentration markedly affected | 173 | 5.8 |  |
|  | 4 = overwhelmingly, unable to think of anything else | 62 | 2.1 |  |
|  | Missing | 67 | 2.2 |  |
| **IPOS feeling anxious rating** | 0 = not at all, no effect | 1742 | 58.1 |  |
|  | 1 = slightly, but not bothered to be rid of it | 677 | 22.6 |  |
|  | 2 = moderately, limits some activity or concentration | 339 | 11.3 |  |
|  | 3 = severely, activities or concentration markedly affected | 116 | 3.9 |  |
|  | 4 = overwhelmingly, unable to think of anything else | 56 | 1.9 |  |
|  | Missing | 66 | 2.2 |  |
| **IPOS feeling depressed rating** | 0 = not at all, no effect | 2014 | 67.2 |  |
|  | 1 = slightly, but not bothered to be rid of it | 467 | 15.6 |  |
|  | 2 = moderately, limits some activity or concentration | 283 | 9.4 |  |
|  | 3 = severely, activities or concentration markedly affected | 112 | 3.7 |  |
|  | 4 = overwhelmingly, unable to think of anything else | 54 | 1.8 |  |
|  | Missing | 66 | 2.2 |  |
| **IPOS changes in skin rating** | 0 = not at all, no effect | 2031 | 67.8 |  |
|  | 1 = slightly, but not bothered to be rid of it | 514 | 17.2 |  |
|  | 2 = moderately, limits some activity or concentration | 247 | 8.2 |  |
|  | 3 = severely, activities or concentration markedly affected | 88 | 2.9 |  |
|  | 4 = overwhelmingly, unable to think of anything else | 32 | 1.1 |  |
|  | Missing | 84 | 2.8 |  |
| **IPOS diarrhoea rating** | 0 = not at all, no effect | 2371 | 79.1 |  |
|  | 1 = slightly, but not bothered to be rid of it | 325 | 10.8 |  |
|  | 2 = moderately, limits some activity or concentration | 141 | 4.7 |  |
|  | 3 = severely, activities or concentration markedly affected | 54 | 1.8 |  |
|  | 4 = overwhelmingly, unable to think of anything else | 26 | 0.9 |  |
|  | Missing | 79 | 2.6 |  |
| **Hospital Anxiety and Depression Scale (HADS) anxiety significant** | No | 2283 | 76.2 |  |
|  | Yes | 713 | 23.8 | Significance defined as a score of 8 or above |
| **Hospital Anxiety and Depression Scale (HADS) depression significant** | No | 2431 | 81.1 |  |
|  | Yes | 565 | 18.9 | Significance defined as a score of 8 or above |
| **Six Item Cognitive Impairment Test (6CIT) significant** | No | 2756 | 92.0 |  |
|  | Yes | 240 | 8.0 | Significance defined as a score of 8 or above |
| **Six Item Cognitive Impairment Test (6CIT) levels** | No issues | 2733 | 91.2 |  |
|  | Score 8-9 | 110 | 3.7 |  |
|  | Score 10 or above | 130 | 4.3 |  |
|  | Missing | 23 | 0.8 |  |
| **EQ-5D-5L mobility dimension** | 1 = I have no problems in walking about | 1493 | 49.8 |  |
|  | 2 = I have slight problems in walking about | 564 | 18.8 |  |
|  | 3 = I have moderate problems in walking about | 541 | 18.1 |  |
|  | 4 = I have severe problems in walking about | 341 | 11.4 |  |
|  | 5 = I am unable to walk about | 19 | 0.6 |  |
|  | Missing | 38 | 1.3 |  |
| **EQ-5D-5L mobility dimension - score of 2 or above** | No | 1531 | 51.1 |  |
|  | Yes | 1465 | 48.9 |  |
| **EQ-5D-5L self-care dimension** | 1 = I have no problems washing or dressing myself | 2410 | 80.4 |  |
|  | 2 = I have slight problems washing or dressing myself | 277 | 9.2 |  |
|  | 3 = I have moderate problems washing or dressing myself | 202 | 6.7 |  |
|  | 4 = I have severe problems washing or dressing myself | 62 | 2.1 |  |
|  | 5 = I am unable to wash or dress myself | 7 | 0.2 |  |
|  | Missing | 38 | 1.3 |  |
| **EQ-5D-5L self-care - score of 2 or above** | No | 2448 | 81.7 |  |
|  | Yes | 548 | 18.3 |  |
| **EQ-5D-5L usual activities dimension** | 1 = I have no problems doing my usual activities | 1634 | 54.5 |  |
|  | 2 = I have slight problems doing my usual activities | 592 | 19.8 |  |
|  | 3 = I have moderate problems doing my usual activities | 448 | 15.0 |  |
|  | 4 = I have severe problems doing my usual activities | 221 | 7.4 |  |
|  | 5 = I am unable to do my usual activities | 63 | 2.1 |  |
|  | Missing | 38 | 1.3 |  |
| **EQ-5D-5L usual activities - score of 2 or above** | No | 1672 | 55.8 |  |
|  | Yes | 1324 | 44.2 |  |
| **EQ-5D-5L pain or discomfort dimension** | 1 = I have no pain or discomfort | 1185 | 39.6 |  |
|  | 2 = I have slight pain or discomfort | 804 | 26.8 |  |
|  | 3 = I have moderate pain or discomfort | 654 | 21.8 |  |
|  | 4 = I have severe pain or discomfort | 277 | 9.2 |  |
|  | 5 = I have extreme pain or discomfort | 38 | 1.3 |  |
|  | Missing | 38 | 1.3 |  |
| **EQ-5D-5L pain or discomfort - score of 2 or above** | No | 1223 | 40.8 |  |
|  | Yes | 1773 | 59.2 |  |
| **EQ-5D-5L anxiety and depression dimension** | 1 = I am not anxious or depressed | 1944 | 64.9 |  |
|  | 2 = I am slightly anxious or depressed | 628 | 21.0 |  |
|  | 3 = I am moderately anxious or depressed | 303 | 10.1 |  |
|  | 4 = I am severely anxious or depressed | 54 | 1.8 |  |
|  | 5 = I am extremely anxious or depressed | 29 | 1.0 |  |
|  | Missing | 38 | 1.3 |  |
| **EQ-5D-5L anxiety and depression - score of 2 or above** | No | 1982 | 66.2 |  |
|  | Yes | 1014 | 33.8 |  |
| **Number of dimensions with problems** | **0** | 757 | 25.3 |  |
|  | **1** | 540 | 18 |  |
|  | **2** | 457 | 15.3 |  |
|  | **3** | 468 | 15.6 |  |
|  | **4** | 414 | 13.8 |  |
|  | **5** | 322 | 10.7 |  |
|  | **Missing** | 38 | 1.3 |  |
| **EQ-5D-3L index value less than 0.0** | No | 2942 | 98.2 |  |
|  | Yes | 54 | 1.8 |  |
| **EQ-5D-5L index value less than 0.0** | No | 2979 | 99.4 |  |
|  | Yes | 17 | 0.6 |  |
| **EQ-5D-5L problems in any dimension** | No | 795 | 26.5 |  |
|  | Yes | 2201 | 73.5 | Positive if index value is less than 1 (a health state other than 11111) |
| *Minor discrepancies in fields such as diagnosis/ethnicity may be seen between this table and the Taal et al 2020 baseline paper - this is due to minor differences in classification* | | | | |

## Table S2. Descriptors of continuous variables in entire cohort

|  | **Mean** | **SD** | **Minimum** | **Lower quartile** | **Median** | **Upper quartile** | **Maximum** | **Interquartile range** | **n missing** | **percent missing** | **Notes** |
| --- | --- | --- | --- | --- | --- | --- | --- | --- | --- | --- | --- |
| **Age at baseline** | 62.6 | 14.8 | 18.0 | 53.0 | 66.0 | 74.0 | 95.0 | 21.0 | 0.0 | 0.0 |  |
| **Number of comorbidities** | 3.5 | 2.2 | 1.0 | 2.0 | 3.0 | 5.0 | 19.0 | 3.0 | 0.0 | 0.0 |  |
| **Charlson Comorbidity Index score** | 3.4 | 2.2 | 0.0 | 2.0 | 3.0 | 5.0 | 11.0 | 3.0 | 0.0 | 0.0 |  |
| **Number of cigarettes per day** | 1.0 | 4.1 | 0.0 | 0.0 | 0.0 | 0.0 | 58.0 | 0.0 | 0.0 | 0.0 |  |
| **Units of alcohol per week** | 5.5 | 10.2 | 0.0 | 0.0 | 1.0 | 8.0 | 184.0 | 8.0 | 98.0 | 3.3 |  |
| **Number of regular medications** | 7.8 | 4.5 | 1.0 | 4.0 | 7.0 | 11.0 | 33.0 | 7.0 | 0.0 | 0.0 |  |
| **Body Mass Index (kg/m^2^)** | 29.6 | 6.3 | 14.1 | 25.3 | 28.7 | 33.0 | 76.9 | 7.7 | 81.0 | 2.7 |  |
| **Karnofsky performance status** | 86.5 | 15.6 | 0.0 | 80.0 | 90.0 | 100.0 | 100.0 | 20.0 | 19.0 | 0.6 |  |
| **Hip circumference (cm)** | 107.6 | 13.6 | 36.0 | 100.0 | 106.0 | 114.5 | 171.0 | 14.5 | 46.0 | 1.5 |  |
| **Waist circumference (cm)** | 101.7 | 16.6 | 30.5 | 91.5 | 101.2 | 112.0 | 179.2 | 20.5 | 41.0 | 1.4 |  |
| **Waist hip ratio** | 0.9 | 0.1 | 0.3 | 0.9 | 1.0 | 1.0 | 2.5 | 0.1 | 53.0 | 1.8 |  |
| **Waist height ratio** | 0.6 | 0.1 | 0.2 | 0.5 | 0.6 | 0.7 | 1.0 | 0.1 | 78.0 | 2.6 |  |
| **Mean systolic BP (mmHg)** | 139.3 | 20.4 | 70.0 | 125.0 | 137.0 | 151.3 | 228.0 | 26.3 | 4.0 | 0.1 |  |
| **Mean diastolic BP (mmHg)** | 79.9 | 12.4 | 22.0 | 72.0 | 79.0 | 88.0 | 153.0 | 16.0 | 4.0 | 0.1 |  |
| **Mean MAP (mmHg)** | 99.7 | 12.9 | 50.7 | 91.3 | 99.0 | 107.3 | 173.0 | 16.0 | 4.0 | 0.1 |  |
| **Timed up and go result (seconds)** | 11.2 | 6.4 | 1.0 | 7.8 | 9.5 | 12.2 | 100.0 | 4.3 | 190.0 | 6.3 |  |
| **Best hand grip strength (kg)** | 27.6 | 11.0 | 5.3 | 19.4 | 26.6 | 34.9 | 73.2 | 15.5 | 72.0 | 2.4 |  |
| **Number of admissions in the last year** | 0.4 | 0.9 | 0.0 | 0.0 | 0.0 | 1.0 | 11.0 | 1.0 | 0.0 | 0.0 |  |
| **Number of days admitted in the last year** | 3.2 | 10.6 | 0.0 | 0.0 | 0.0 | 1.0 | 190.0 | 1.0 | 0.0 | 0.0 |  |
| **Urine albumin creatinine ratio (uACR) local laboratory (mg/mmol)** | 82.3 | 155.1 | 0.1 | 4.3 | 22.7 | 95.1 | 3000.0 | 90.8 | 653.0 | 21.8 |  |
| **Albumin serum local laboratory (g/L)** | 40.5 | 5.3 | 10.0 | 37.0 | 41.0 | 44.0 | 59.0 | 7.0 | 191.0 | 6.4 |  |
| **Total cholesterol local laboratory (mmol/L)** | 4.7 | 1.3 | 1.3 | 3.8 | 4.6 | 5.5 | 12.2 | 1.7 | 452.0 | 15.1 |  |
| **Triglycerides local laboratory (mmol/L)** | 2.0 | 1.4 | 0.2 | 1.2 | 1.6 | 2.4 | 26.0 | 1.2 | 444.0 | 14.8 |  |
| **HDL local laboratory (mmol/L)** | 1.4 | 0.5 | 0.4 | 1.1 | 1.3 | 1.7 | 5.2 | 0.6 | 446.0 | 14.9 |  |
| **LDL local laboratory (mmol/L)** | 2.5 | 1.1 | 0.1 | 1.7 | 2.4 | 3.1 | 8.0 | 1.4 | 1732.0 | 57.8 |  |
| **Sodium local laboratory (mmol/L)** | 139.7 | 3.1 | 125.0 | 138.0 | 140.0 | 142.0 | 151.0 | 4.0 | 183.0 | 6.1 |  |
| **Potassium local laboratory (mmol/L)** | 4.6 | 0.5 | 2.4 | 4.3 | 4.6 | 5.0 | 6.7 | 0.7 | 198.0 | 6.6 |  |
| **Bicarbonate local laboratory (mmol/L)** | 24.6 | 3.4 | 9.0 | 22.2 | 24.7 | 27.0 | 38.0 | 4.8 | 357.0 | 11.9 |  |
| **Urea local laboratory (mmol/L)** | 12.9 | 6.0 | 2.7 | 8.6 | 11.6 | 15.9 | 67.0 | 7.3 | 193.0 | 6.4 |  |
| **Creatinine serum local laboratory (umol/L)** | 177.0 | 70.3 | 42.0 | 124.8 | 164.0 | 214.0 | 581.0 | 89.3 | 172.0 | 5.7 |  |
| **eGFR local laboratory (ml/min/1.73m²)** | 35.6 | 14.6 | 7.0 | 24.0 | 33.0 | 45.0 | 129.0 | 21.0 | 281.0 | 9.4 |  |
| **C-reactive protein (CRP) local laboratory (mg/L)** | 8.2 | 15.6 | 0.0 | 2.0 | 5.0 | 8.0 | 205.0 | 6.0 | 1029.0 | 34.3 |  |
| **HbA1c local laboratory (mmol/mol)** | 50.5 | 18.6 | 4.1 | 39.0 | 47.0 | 60.8 | 137.2 | 21.8 | 2016.0 | 67.3 |  |
| **Haemoglobin local laboratory (g/dL)** | 126.8 | 18.1 | 36.0 | 114.0 | 126.0 | 139.0 | 189.0 | 25.0 | 198.0 | 6.6 |  |
| **Total white blood cell count local laboratory (10⁹/L)** | 7.6 | 9.9 | 0.3 | 5.8 | 7.1 | 8.7 | 514.0 | 2.9 | 200.0 | 6.7 |  |
| **Platelets local laboratory (10⁹/L)** | 240.2 | 70.0 | 19.0 | 193.0 | 232.0 | 282.0 | 615.0 | 89.0 | 205.0 | 6.8 |  |
| **Phosphate local laboratory (mmol/L)** | 1.1 | 0.2 | 0.5 | 1.0 | 1.1 | 1.3 | 2.3 | 0.3 | 232.0 | 7.7 |  |
| **Parathyroid hormone (PTH) local laboratory (pmol/L)** | 45.9 | 76.4 | 0.9 | 7.1 | 15.7 | 56.0 | 1479.0 | 48.9 | 502.0 | 16.8 |  |
| **Haematocrit local laboratory (L/L)** | 7.1 | 46.8 | 0.1 | 0.4 | 0.4 | 0.4 | 718.0 | 0.1 | 181.0 | 6.0 |  |
| **Urate local laboratory (umol/L)** | 420.4 | 115.3 | 0.5 | 348.0 | 420.0 | 491.5 | 1162.0 | 143.5 | 1541.0 | 51.4 |  |
| **Calcium adjusted local laboratory (mmol/L)** | 2.4 | 0.1 | 1.7 | 2.3 | 2.4 | 2.4 | 2.9 | 0.1 | 272.0 | 9.1 |  |
| **Calcium unadjusted local laboratory (mmol/L)** | 2.3 | 0.1 | 1.4 | 2.3 | 2.4 | 2.4 | 2.8 | 0.2 | 973.0 | 32.5 |  |
| **Urine protein creatinine ratio (uPCR) local laboratory (mg/mmol)** | 134.7 | 250.9 | 0.0 | 16.0 | 48.2 | 150.0 | 5354.0 | 134.0 | 998.0 | 33.3 |  |
| **N-terminal pro-B-type natriuretic peptide (BNP) central laboratory (ng/L)** | 940.6 | 2775.1 | 5.0 | 101.0 | 249.5 | 708.0 | 63371.0 | 607.0 | 58.0 | 1.9 |  |
| **Creatinine serum central laboratory (umol/L)** | 176.7 | 70.5 | 43.0 | 125.0 | 163.0 | 215.0 | 607.0 | 90.0 | 57.0 | 1.9 |  |
| **C-reactive protein (CRP) central laboratory (mg/L)** | 5.3 | 10.2 | 0.3 | 1.1 | 2.6 | 5.7 | 172.7 | 4.6 | 67.0 | 2.2 |  |
| **Growth/differentiation factor 15 (GDF15) central laboratory (pg/ml)** | 3249.4 | 3842.3 | 400.0 | 1605.0 | 2503.0 | 3851.0 | 100000.0 | 2246.0 | 67.0 | 2.2 |  |
| **Troponin central laboratory (ng/L)** | 26.3 | 36.8 | 3.0 | 9.4 | 17.2 | 31.5 | 1043.0 | 22.1 | 58.0 | 1.9 |  |
| **uACR central laboratory (mg/g)** | 798.3 | 2254.4 | 1.0 | 33.0 | 208.5 | 925.8 | 97262.0 | 892.8 | 270.0 | 9.0 |  |
| **eGFR (CKD EPI formula) central laboratory (ml/min/1.73m²)** | 37.3 | 17.9 | 6.0 | 24.0 | 34.0 | 47.0 | 134.0 | 23.0 | 0.0 | 0.0 |  |
| **Integrated Palliative care outcome scale (IPOS) total score** | 11.6 | 9.8 | 0.0 | 4.0 | 9.0 | 16.0 | 62.0 | 12.0 | 33.0 | 1.1 |  |
| **Hospital Anxiety and Depression Scale (HADS) overall anxiety score** | 5.0 | 4.1 | 0.0 | 2.0 | 4.0 | 7.0 | 20.0 | 5.0 | 33.0 | 1.1 |  |
| **Hospital Anxiety and Depression Scale (HADS) overall depression score** | 4.4 | 3.6 | 0.0 | 1.0 | 4.0 | 6.0 | 20.0 | 5.0 | 33.0 | 1.1 |  |
| **Hospital Anxiety and Depression Scale (HADS) total score** | 9.4 | 6.8 | 0.0 | 4.0 | 8.0 | 13.0 | 39.0 | 9.0 | 33.0 | 1.1 |  |
| **Six Item Cognitive Impairment Test (6CIT) overall score** | 2.4 | 3.1 | 0.0 | 0.0 | 2.0 | 4.0 | 28.0 | 4.0 | 23.0 | 0.8 |  |
| **EQ-5D-5L health rating (visual analogue scale)** | 71.24 | 20.21 | 0.00 | 60.00 | 75.00 | 88.00 | 100.00 | 28.00 | 38.00 | 1.27 |  |
| **EQ-5D-5L index value** | 0.80 | 0.23 | -0.16 | 0.72 | 0.86 | 1.00 | 1.00 | 0.28 | 38.00 | 1.27 | Indexed using Devlin et al. UK dataset |
| **EQ-5D-3L mapped index value** | 0.73 | 0.26 | -0.29 | 0.63 | 0.79 | 0.99 | 0.99 | 0.36 | 38.00 | 1.27 | Mapped from 5L questionnaire values using Hernandez-Alvara et al. method |

## Table S3. Multivariable mixed effects regression models of health-related quality of life outcome measures

|  | **EQ-5D-3L mapped index value linear regression** | | | **EQ-5D-5L health rating (visual analogue scale) linear regression** | | | **EQ-5D-5L problems in any dimension logistic regression** | | | **Number of dimensions in EQ-5D-5L scored 2 or above - linear regression** | | | ***n (%) in cohort with complete EQ-5D-5L data*** |  |
| --- | --- | --- | --- | --- | --- | --- | --- | --- | --- | --- | --- | --- | --- | --- |
|  |  |  |  |  |  |  |  |  |  |  |  |  |  |  |
| *Risk factors* | *Coefficient B* | ***95% Confidence intervals*** | *p-values* | *Coefficient B* | ***95% Confidence intervals*** | *p-values* | *Odds Ratios* | ***95% Confidence intervals*** | *p-values* | *Coefficient B* | ***95% Confidence intervals*** | *p-values* |  |  |
| (Intercept) | 0.955 | 0.780 to 1.130 | **<0.001** | 73.438 | 57.852 to 89.025 | **<0.001** | 13.107 | 0.624 to 275.371 | **0.098** | 1.085 | -0.038 to 2.209 | 0.058 | - |  |
| Age | 0.000 | -0.001 to 0.000 | 0.372 | 0.093 | 0.032 to 0.153 | **0.003** | 1.007 | 0.997 to 1.018 | 0.181 | 0.005 | 0.001 to 0.009 | **0.023** | - |  |
| Sex (female) | -0.031 | -0.047 to -0.015 | **<0.001** | -0.355 | -1.837 to 1.127 | 0.638 | 1.245 | 0.948 to 1.634 | 0.115 | 0.163 | 0.059 to 0.268 | **0.002** | 1229 (41.5%) |  |
| Ethnicity [Asian] | -0.014 | -0.045 to 0.018 | 0.398 | 0.055 | -2.801 to 2.910 | 0.970 | 0.649 | 0.391 to 1.078 | 0.095 | -0.023 | -0.226 to 0.180 | 0.825 | 197 (6.7%) |  |
| Ethnicity [Black] | 0.000 | -0.048 to 0.047 | 0.995 | 0.377 | -3.907 to 4.662 | 0.863 | 1.071 | 0.482 to 2.379 | 0.867 | -0.048 | -0.352 to 0.256 | 0.757 | 89 (3.0%) |  |
| Ethnicity [Mixed] | 0.008 | -0.065 to 0.082 | 0.826 | 2.369 | -4.313 to 9.051 | 0.487 | 2.171 | 0.612 to 7.703 | 0.230 | 0.224 | -0.247 to 0.696 | 0.351 | 31 (1.0%) |  |
| Ethnicity [Other] | 0.061 | -0.003 to 0.126 | **0.064** | 2.450 | -3.397 to 8.297 | 0.411 | 0.846 | 0.333 to 2.148 | 0.724 | -0.443 | -0.860 to -0.026 | **0.037** | 53 (1.8%) |  |
| Index of Multiple Deprivation (IMD) quintile 2 | 0.012 | -0.012 to 0.035 | 0.329 | 0.624 | -1.515 to 2.763 | 0.567 | 0.777 | 0.539 to 1.120 | 0.176 | -0.037 | -0.188 to 0.113 | 0.628 | 612 (20.7%) |  |
| IMD quintile 3 | -0.010 | -0.034 to 0.014 | 0.405 | 1.305 | -0.894 to 3.503 | 0.245 | 1.185 | 0.796 to 1.764 | 0.402 | 0.034 | -0.121 to 0.189 | 0.665 | 549 (18.6%) |  |
| IMD quintile 4 | -0.009 | -0.034 to 0.016 | 0.481 | -1.109 | -3.389 to 1.171 | 0.340 | 0.972 | 0.644 to 1.468 | 0.894 | 0.061 | -0.100 to 0.222 | 0.456 | 543 (18.4%) |  |
| IMD quintile 5 | -0.035 | -0.060 to -0.010 | **0.006** | -1.237 | -3.523 to 1.048 | 0.289 | 1.016 | 0.662 to 1.561 | 0.941 | 0.131 | -0.031 to 0.293 | 0.113 | 607 (20.6%) |  |
| Education [GCSE/NVQ/A-level] | 0.020 | -0.000 to 0.040 | **0.050** | 1.488 | -0.313 to 3.288 | 0.105 | 0.911 | 0.638 to 1.301 | 0.609 | -0.090 | -0.218 to 0.037 | 0.163 | 1347 (45.5%) |  |
| Education [Higher education] | 0.016 | -0.007 to 0.039 | 0.175 | 0.980 | -1.125 to 3.086 | 0.361 | 0.789 | 0.534 to 1.165 | 0.234 | -0.098 | -0.247 to 0.051 | 0.199 | 782 (26.4%) |  |
| eGFR (ml/min/1.73m^2^) | 0.000 | -0.000 to 0.001 | 0.835 | 0.071 | 0.027 to 0.116 | **0.002** | 0.995 | 0.987 to 1.003 | 0.212 | -0.002 | -0.006 to 0.001 | 0.130 | - |  |
| Number of comorbidities | -0.015 | -0.019 to -0.011 | **<0.001** | -1.113 | -1.485 to -0.742 | **<0.001** | 1.107 | 1.023 to 1.199 | **0.012** | 0.066 | 0.040 to 0.093 | **<0.001** | - |  |
| 6-item Cognitive Impairment Test (6CIT) score 8 or above | -0.062 | -0.090 to -0.034 | **<0.001** | -1.895 | -4.413 to 0.622 | 0.140 | 2.812 | 1.592 to 4.964 | **<0.001** | 0.393 | 0.215 to 0.571 | **<0.001** | 237 (8.1%) |  |
| BMI [Underweight] | 0.023 | -0.057 to 0.103 | 0.570 | -4.301 | -11.595 to 2.993 | 0.248 | 1.171 | 0.290 to 4.723 | 0.825 | -0.165 | -0.678 to 0.349 | 0.529 | 32 (1.1%) |  |
| BMI [Overweight] | -0.001 | -0.022 to 0.020 | 0.916 | -0.259 | -2.150 to 1.632 | 0.788 | 0.976 | 0.703 to 1.357 | 0.887 | -0.035 | -0.168 to 0.099 | 0.609 | 1016 (35.3%) |  |
| Body Mass Index (BMI, kg/m^2^) [Obese] | -0.037 | -0.058 to -0.016 | **0.001** | -2.747 | -4.635 to -0.858 | **0.004** | 1.202 | 0.856 to 1.687 | 0.288 | 0.202 | 0.069 to 0.335 | **0.003** | 1190 (41.3%) |  |
| Smoking status [Ex-smoker] | -0.017 | -0.033 to -0.001 | **0.043** | -0.181 | -1.680 to 1.318 | 0.813 | 1.124 | 0.857 to 1.475 | 0.397 | 0.122 | 0.016 to 0.228 | **0.024** | 1205 (40.9%) |  |
| Smoking status [Current smoker] | -0.004 | -0.033 to 0.025 | 0.786 | 0.832 | -1.803 to 3.466 | 0.536 | 1.135 | 0.666 to 1.934 | 0.641 | 0.075 | -0.111 to 0.260 | 0.430 | 263 (8.9%) |  |
| Sarcopenia present | -0.062 | -0.080 to -0.043 | **<0.001** | -4.083 | -5.774 to -2.392 | **<0.001** | 1.859 | 1.322 to 2.615 | **<0.001** | 0.361 | 0.242 to 0.481 | **<0.001** | 889 (30.1%) |  |
| Prednisolone | 0.011 | -0.014 to 0.036 | 0.404 | -1.466 | -3.736 to 0.804 | 0.205 | 0.830 | 0.540 to 1.275 | 0.395 | 0.113 | -0.047 to 0.273 | 0.167 | 354 (12.0%) |  |
| Renin-angiotensin system inhibitors | 0.011 | -0.006 to 0.028 | 0.200 | 0.038 | -1.486 to 1.563 | 0.961 | 0.863 | 0.650 to 1.144 | 0.305 | -0.104 | -0.212 to 0.003 | **0.057** | 1954 (66.1%) |  |
| On 10 or more regular medications | -0.065 | -0.085 to -0.046 | **<0.001** | -1.717 | -3.484 to 0.049 | 0.057 | 1.382 | 0.970 to 1.969 | 0.073 | 0.356 | 0.231 to 0.481 | **<0.001** | 948 (32.0%) |  |
| Hospital Anxiety and Depression Scale (HADS) depression score 8 or above | -0.159 | -0.182 to -0.137 | **<0.001** | -13.231 | -15.296 to -11.165 | **<0.001** | 3.506 | 1.899 to 6.474 | **<0.001** | 0.934 | 0.788 to 1.080 | **<0.001** | 563 (19.0%) |  |
| HADS anxiety score 8 or above | -0.090 | -0.110 to -0.069 | **<0.001** | -4.965 | -6.842 to -3.087 | **<0.001** | 3.022 | 1.981 to 4.611 | **<0.001** | 0.594 | 0.462 to 0.727 | **<0.001** | 709 (24.0%) |  |
| Integrated Palliative care Outcome Scale (IPOS) pain symptoms present | -0.134 | -0.152 to -0.117 | **<0.001** | -4.004 | -5.602 to -2.406 | **<0.001** | 7.001 | 5.380 to 9.112 | **<0.001** | 1.037 | 0.925 to 1.150 | **<0.001** | 1848 (63.8%) |  |
| IPOS shortness of breath symptoms present | -0.026 | -0.043 to -0.008 | **0.005** | -2.953 | -4.576 to -1.330 | **<0.001** | 1.639 | 1.236 to 2.173 | **0.001** | 0.328 | 0.213 to 0.442 | **<0.001** | 1622 (56.2%) |  |
| IPOS weakness or lack of energy symptoms present | -0.034 | -0.054 to -0.014 | **0.001** | -7.477 | -9.282 to -5.672 | **<0.001** | 2.516 | 1.904 to 3.325 | **<0.001** | 0.421 | 0.293 to 0.548 | **<0.001** | 2125 (72.5%) |  |
| Haemoglobin less than 100 g/dL | -0.047 | -0.085 to -0.010 | **0.012** | -3.592 | -6.968 to -0.217 | **0.037** | 1.239 | 0.586 to 2.622 | 0.575 | 0.243 | 0.005 to 0.482 | 0.045 | 147 (5.0%) |  |
| Phosphate greater than 1.5 mmol/L | -0.007 | -0.047 to 0.032 | 0.716 | 2.542 | -1.041 to 6.125 | 0.164 | 0.944 | 0.458 to 1.943 | 0.875 | 0.039 | -0.213 to 0.292 | 0.760 | 121 (4.1%) |  |
| Parathyroid hormone (PTH) Raised (7.2-15.7 pmol/L) | 0.005 | -0.018 to 0.028 | 0.668 | 1.590 | -0.455 to 3.634 | 0.127 | 0.867 | 0.600 to 1.254 | 0.448 | -0.052 | -0.198 to 0.093 | 0.482 | 618 (25.1%) |  |
| PTH High (15.8-56.0 pmol/L) | 0.017 | -0.008 to 0.042 | 0.190 | 3.127 | 1.113 to 5.141 | **0.002** | 0.746 | 0.495 to 1.123 | 0.160 | -0.250 | -0.411 to -0.088 | **0.003** | 626 (25.4%) |  |
| PTH Very high (>56.0 pmol/L) | 0.003 | -0.028 to 0.034 | 0.841 | -0.661 | -2.801 to 1.480 | 0.545 | 0.900 | 0.547 to 1.482 | 0.678 | -0.093 | -0.293 to 0.107 | 0.364 | 590 (24.0%) |  |
| Calcium (adjusted, mmol/L) | 0.039 | -0.030 to 0.108 | 0.270 | 3.326 | -2.868 to 9.519 | 0.293 | 0.166 | 0.049 to 0.560 | **0.004** | -0.525 | -0.970 to -0.081 | **0.021** | - |  |
| **Random Effects** | | | | | | | | | | | | |  |  |
| **σ^2^** | 0.03 | | | 265.22 | | | 3.29 | | | 1.33 | | |  |  |
| **τ_00_** | 0.00 recruited_group | | | 0.00 recruited_group | | | 0.08 recruited_group | | | 0.02 recruited_group | | |  |  |
| **ICC** | 0.01 | | |  | | | 0.02 | | | 0.01 | | |  |  |
| **N** | 9 recruited_group | | | 9 recruited_group | | | 9 recruited_group | | | 9 recruited_group | | |  |  |
| **Observations** | 2266 | | | 2266 | | | 2266 | | | 2266 | | |  |  |
| **Marginal R^2^ / Conditional R^2^** | 0.507 / 0.513 | | | 0.358 / NA | | | 0.560 / 0.570 | | | 0.539 / 0.544 | | |  |  |
| **AIC** | -1058.718 | | | 19066.717 | | | 1749.233 | | | 7267.92 | | |  |  |
| p-values in bold denote statistical significance of <0.05 Sex reference category = Male, Ethnicity reference category = White, IMD reference category = quintile 1 (most deprived), Education reference category = None (below GCSE level), BMI reference category = Normal weight (BMI 18.5 to 24.9 kg/m2) Smoking status reference category = non-smoker, PTH reference category = PTH low to normal (0 to 7.1 pmol/L) Models adjusted for recruitment region as a random effect | | | | | | | | | | | | | |  |

##

## Table S4. Multivariable mixed effects regression models of problems in each EQ-5D-5L dimension

|  | **Problems reported in EQ-5D-5L Mobility dimension logistic regression** | | | **Problems reported in EQ-5D-5L Self-care dimension logistic regression** | | | **Problems reported in EQ-5D-5L Usual activities dimension logistic regression** | | | **Problems reported in EQ-5D-5L pain/discomfort dimension logistic regression** | | | **Problems reported in EQ-5D-5L anxiety/depression dimension logistic regression** | | |  |
| --- | --- | --- | --- | --- | --- | --- | --- | --- | --- | --- | --- | --- | --- | --- | --- | --- |
|  |  |  |  |  |  |  |  |  |  |  |  |  |  |  |  |  |
| ***Risk factors*** | ***Odds Ratios*** | ***95% Confidence intervals*** | ***p-values*** | ***Odds Ratios*** | ***95% Confidence intervals*** | ***p-values*** | ***Odds Ratios*** | ***95% Confidence intervals*** | ***p-values*** | ***Odds Ratios*** | ***95% Confidence intervals*** | ***p-values*** | ***Odds Ratios*** | ***95% Confidence intervals*** | ***p-values*** |  |
| (Intercept) | 0.070 | 0.006 to 0.860 | **0.038** | 0.005 | 0.000 to 0.120 | **0.001** | 0.264 | 0.023 to 3.023 | 0.285 | 0.459 | 0.028 to 7.472 | 0.585 | 0.703 | 0.056 to 8.864 | 0.785 |  |
| Age | 1.035 | 1.025 to 1.046 | **<0.001** | 1.005 | 0.992 to 1.018 | 0.456 | 1.010 | 1.000 to 1.020 | **0.045** | 1.000 | 0.989 to 1.010 | 0.961 | 0.991 | 0.981 to 1.000 | 0.059 |  |
| Sex (female) | 1.193 | 0.943 to 1.508 | 0.140 | 1.287 | 0.958 to 1.728 | 0.093 | 1.388 | 1.106 to 1.743 | **0.005** | 1.448 | 1.121 to 1.870 | **0.005** | 1.251 | 0.992 to 1.577 | 0.059 |  |
| Ethnicity [Asian] | 1.039 | 0.657 to 1.643 | 0.869 | 1.514 | 0.850 to 2.697 | 0.159 | 0.838 | 0.531 to 1.323 | 0.448 | 0.889 | 0.541 to 1.461 | 0.642 | 0.967 | 0.610 to 1.533 | 0.886 |  |
| Ethnicity [Black] | 0.662 | 0.329 to 1.328 | 0.245 | 0.881 | 0.365 to 2.128 | 0.778 | 0.869 | 0.440 to 1.716 | 0.686 | 1.931 | 0.889 to 4.195 | 0.096 | 0.740 | 0.361 to 1.518 | 0.412 |  |
| Ethnicity [Mixed] | 1.471 | 0.544 to 3.977 | 0.447 | 1.563 | 0.435 to 5.614 | 0.494 | 1.499 | 0.543 to 4.137 | 0.434 | 1.656 | 0.511 to 5.372 | 0.400 | 1.294 | 0.437 to 3.832 | 0.642 |  |
| Ethnicity [Other] | 0.162 | 0.045 to 0.591 | **0.006** | 0.199 | 0.019 to 2.043 | 0.174 | 0.253 | 0.070 to 0.913 | **0.036** | 0.739 | 0.275 to 1.988 | 0.550 | 1.276 | 0.505 to 3.222 | 0.606 |  |
| Number of comorbidities | 1.130 | 1.065 to 1.200 | **<0.001** | 1.151 | 1.080 to 1.228 | **<0.001** | 1.112 | 1.050 to 1.176 | **<0.001** | 1.101 | 1.028 to 1.179 | **0.006** | 1.006 | 0.949 to 1.066 | 0.840 |  |
| 6-item Cognitive Impairment Test (6CIT) score 8 or above | 2.123 | 1.396 to 3.228 | **<0.001** | 1.973 | 1.275 to 3.053 | **0.002** | 1.718 | 1.164 to 2.535 | **0.006** | 1.389 | 0.889 to 2.170 | 0.149 | 1.795 | 1.225 to 2.631 | **0.003** |  |
| Index of Multiple Deprivation (IMD) quintile 2 | 0.884 | 0.634 to 1.233 | 0.466 | 1.101 | 0.688 to 1.761 | 0.688 | 0.953 | 0.688 to 1.322 | 0.775 | 0.766 | 0.533 to 1.101 | 0.149 | 1.178 | 0.835 to 1.661 | 0.350 |  |
| IMD quintile 3 | 0.993 | 0.700 to 1.409 | 0.970 | 1.142 | 0.719 to 1.813 | 0.574 | 0.898 | 0.640 to 1.260 | 0.534 | 0.934 | 0.640 to 1.362 | 0.722 | 1.202 | 0.848 to 1.705 | 0.302 |  |
| IMD quintile 4 | 1.063 | 0.743 to 1.521 | 0.740 | 1.347 | 0.843 to 2.152 | 0.212 | 0.940 | 0.663 to 1.333 | 0.728 | 1.079 | 0.729 to 1.596 | 0.704 | 1.193 | 0.829 to 1.717 | 0.343 |  |
| IMD quintile 5 | 1.351 | 0.940 to 1.940 | 0.104 | 1.700 | 1.079 to 2.680 | **0.022** | 1.038 | 0.731 to 1.474 | 0.834 | 1.068 | 0.714 to 1.599 | 0.748 | 1.269 | 0.882 to 1.825 | 0.199 |  |
| eGFR (ml/min/1.73m^2^) | 0.996 | 0.988 to 1.003 | 0.235 | 0.998 | 0.988 to 1.007 | 0.619 | 0.992 | 0.984 to 0.999 | **0.022** | 0.992 | 0.984 to 1.000 | **0.047** | 1.004 | 0.997 to 1.011 | 0.276 |  |
| Education [GCSE/NVQ/A-level] | 0.900 | 0.680 to 1.190 | 0.460 | 0.749 | 0.538 to 1.041 | 0.085 | 0.838 | 0.639 to 1.098 | 0.200 | 1.279 | 0.934 to 1.751 | 0.125 | 0.948 | 0.714 to 1.260 | 0.715 |  |
| Education [Higher education] | 0.961 | 0.692 to 1.334 | 0.811 | 0.672 | 0.439 to 1.031 | 0.069 | 0.773 | 0.560 to 1.065 | 0.115 | 1.094 | 0.762 to 1.570 | 0.625 | 0.935 | 0.668 to 1.309 | 0.696 |  |
| BMI [Underweight] | 1.170 | 0.363 to 3.769 | 0.792 | 0.370 | 0.065 to 2.107 | 0.263 | 1.019 | 0.327 to 3.177 | 0.974 | 0.188 | 0.048 to 0.732 | **0.016** | 1.224 | 0.387 to 3.864 | 0.731 |  |
| BMI [Overweight] | 1.105 | 0.818 to 1.494 | 0.516 | 0.873 | 0.578 to 1.319 | 0.518 | 0.966 | 0.719 to 1.297 | 0.817 | 0.973 | 0.704 to 1.345 | 0.870 | 0.776 | 0.574 to 1.049 | 0.099 |  |
| Body Mass Index (BMI, kg/m^2^) [Obese] | 2.172 | 1.613 to 2.925 | **<0.001** | 1.673 | 1.140 to 2.454 | **0.009** | 1.387 | 1.039 to 1.851 | **0.026** | 0.959 | 0.695 to 1.323 | 0.798 | 0.943 | 0.702 to 1.266 | 0.695 |  |
| Hospital Anxiety and Depression Scale (HADS) depression score 8 or above | 2.673 | 1.902 to 3.757 | **<0.001** | 4.531 | 3.298 to 6.224 | **<0.001** | 3.471 | 2.518 to 4.784 | **<0.001** | 2.279 | 1.537 to 3.380 | **<0.001** | 2.754 | 2.038 to 3.722 | **<0.001** |  |
| HADS anxiety score 8 or above | 1.039 | 0.772 to 1.398 | 0.800 | 1.610 | 1.154 to 2.246 | **0.005** | 1.407 | 1.063 to 1.863 | **0.017** | 1.477 | 1.058 to 2.062 | **0.022** | 7.635 | 5.834 to 9.992 | **<0.001** |  |
| On 10 or more regular medications | 1.466 | 1.123 to 1.914 | **0.005** | 1.791 | 1.307 to 2.455 | **<0.001** | 1.521 | 1.175 to 1.968 | **0.001** | 1.260 | 0.927 to 1.712 | 0.140 | 1.891 | 1.442 to 2.480 | **<0.001** |  |
| Sarcopenia present | 2.323 | 1.791 to 3.012 | **<0.001** | 2.474 | 1.832 to 3.343 | **<0.001** | 1.425 | 1.111 to 1.828 | **0.005** | 1.307 | 0.973 to 1.755 | 0.075 | 0.929 | 0.713 to 1.209 | 0.582 |  |
| Integrated Palliative care Outcome Scale (IPOS) pain symptoms present | 3.059 | 2.398 to 3.902 | **<0.001** | 2.639 | 1.775 to 3.924 | **<0.001** | 2.428 | 1.908 to 3.091 | **<0.001** | 19.843 | 15.262 to 25.801 | **<0.001** | 1.381 | 1.072 to 1.779 | **0.013** |  |
| IPOS shortness of breath symptoms present | 1.862 | 1.466 to 2.365 | **<0.001** | 1.544 | 1.098 to 2.172 | **0.012** | 1.933 | 1.532 to 2.441 | **<0.001** | 1.181 | 0.904 to 1.543 | 0.223 | 1.237 | 0.962 to 1.593 | 0.098 |  |
| IPOS weakness or lack of energy symptoms present | 1.813 | 1.370 to 2.399 | **<0.001** | 1.847 | 1.131 to 3.015 | **0.014** | 2.828 | 2.116 to 3.779 | **<0.001** | 1.545 | 1.154 to 2.069 | **0.004** | 1.926 | 1.422 to 2.608 | **<0.001** |  |
| Prednisolone | 1.088 | 0.767 to 1.544 | 0.635 | 1.802 | 1.208 to 2.689 | **0.004** | 1.349 | 0.962 to 1.892 | 0.083 | 1.091 | 0.731 to 1.628 | 0.671 | 0.943 | 0.662 to 1.343 | 0.745 |  |
| Renin-angiotensin system inhibitors | 0.762 | 0.599 to 0.970 | **0.027** | 0.795 | 0.589 to 1.074 | 0.135 | 0.680 | 0.538 to 0.858 | **0.001** | 1.002 | 0.769 to 1.304 | 0.990 | 1.133 | 0.890 to 1.443 | 0.311 |  |
| Smoking status [Ex-smoker] | 1.259 | 0.995 to 1.592 | 0.055 | 1.312 | 0.969 to 1.777 | 0.079 | 1.152 | 0.916 to 1.450 | 0.226 | 1.265 | 0.976 to 1.640 | 0.076 | 1.052 | 0.830 to 1.334 | 0.672 |  |
| Smoking status [Current smoker] | 1.749 | 1.168 to 2.619 | **0.007** | 1.501 | 0.937 to 2.406 | 0.091 | 0.863 | 0.583 to 1.278 | 0.463 | 0.936 | 0.600 to 1.463 | 0.773 | 1.051 | 0.700 to 1.578 | 0.811 |  |
| Haemoglobin less than 100 g/dL | 1.568 | 0.883 to 2.785 | 0.125 | 1.602 | 0.927 to 2.767 | 0.091 | 1.425 | 0.849 to 2.393 | 0.181 | 1.174 | 0.634 to 2.174 | 0.610 | 1.142 | 0.692 to 1.884 | 0.604 |  |
| Phosphate greater than 1.5 mmol/L | 1.107 | 0.613 to 2.000 | 0.735 | 1.035 | 0.550 to 1.946 | 0.915 | 1.129 | 0.643 to 1.983 | 0.673 | 1.042 | 0.543 to 2.001 | 0.901 | 0.981 | 0.566 to 1.701 | 0.945 |  |
| Parathyroid hormone (PTH) Raised (7.2-15.7 pmol/L) | 1.349 | 0.976 to 1.865 | 0.070 | 1.016 | 0.657 to 1.573 | 0.943 | 0.863 | 0.630 to 1.182 | 0.358 | 0.760 | 0.533 to 1.085 | 0.131 | 0.874 | 0.632 to 1.209 | 0.416 |  |
| PTH High (15.8-56.0 pmol/L) | 0.861 | 0.612 to 1.212 | 0.392 | 1.037 | 0.677 to 1.589 | 0.866 | 0.604 | 0.432 to 0.843 | **0.003** | 0.715 | 0.476 to 1.075 | 0.107 | 0.618 | 0.428 to 0.892 | **0.010** |  |
| PTH Very high (>56.0 pmol/L) | 1.121 | 0.760 to 1.653 | 0.563 | 1.539 | 1.000 to 2.366 | **0.050** | 0.759 | 0.516 to 1.118 | 0.163 | 0.652 | 0.395 to 1.078 | 0.095 | 0.774 | 0.492 to 1.218 | 0.268 |  |
| Calcium (adjusted, mmol/L) | 0.362 | 0.133 to 0.980 | **0.046** | 0.801 | 0.242 to 2.655 | 0.717 | 0.501 | 0.192 to 1.306 | 0.157 | 0.510 | 0.168 to 1.544 | 0.233 | 0.457 | 0.169 to 1.237 | 0.123 |  |
| **Random Effects** | | | | | | | | | | | | | | | |  |
| σ^2^ | 3.29 | | | 3.29 | | | 3.29 | | | 3.29 | | | 3.29 | | |  |
| τ_00_ | 0.01 _recruited_group_ | | | 0.00 _recruited_group_ | | | 0.01 _recruited_group_ | | | 0.18 _recruited_group_ | | | 0.10 _recruited_group_ | | |  |
| ICC | 0 | | |  | | | 0 | | | 0.05 | | | 0.03 | | |  |
| N | 9 _recruited_group_ | | | 9 _recruited_group_ | | | 9 _recruited_group_ | | | 9 _recruited_group_ | | | 9 _recruited_group_ | | |  |
| Observations | 2167 | | | 2167 | | | 2167 | | | 2167 | | | 2167 | | |  |
| Marginal R^2^ / Conditional R^2^ | 0.517 / 0.519 | | | 0.533 / NA | | | 0.464 / 0.466 | | | 0.520 / 0.544 | | | 0.390 / 0.409 | | |  |
| AIC | 2088.814 | | | 1405.886 | | | 2184.493 | | | 1860.788 | | | 2121.778 | | |  |
| p-values in bold denote statistical significance of <0.05 Sex reference category = Male, Ethnicity reference category = White, IMD reference category = quintile 1 (most deprived), Education reference category = None (below GCSE level), BMI reference category = Normal weight (BMI 18.5 to 24.9 kg/m2) Smoking status reference category = non-smoker, PTH reference category = PTH low to normal (0 to 7.1 pmol/L). Models adjusted for recruitment region as a random effect | | | | | | | | | | | | | | | |  |

# Figure S1. Direct acyclic graph depicting relationships between variables in dataset and outcome of interest, health-related quality of life (HRQoL)


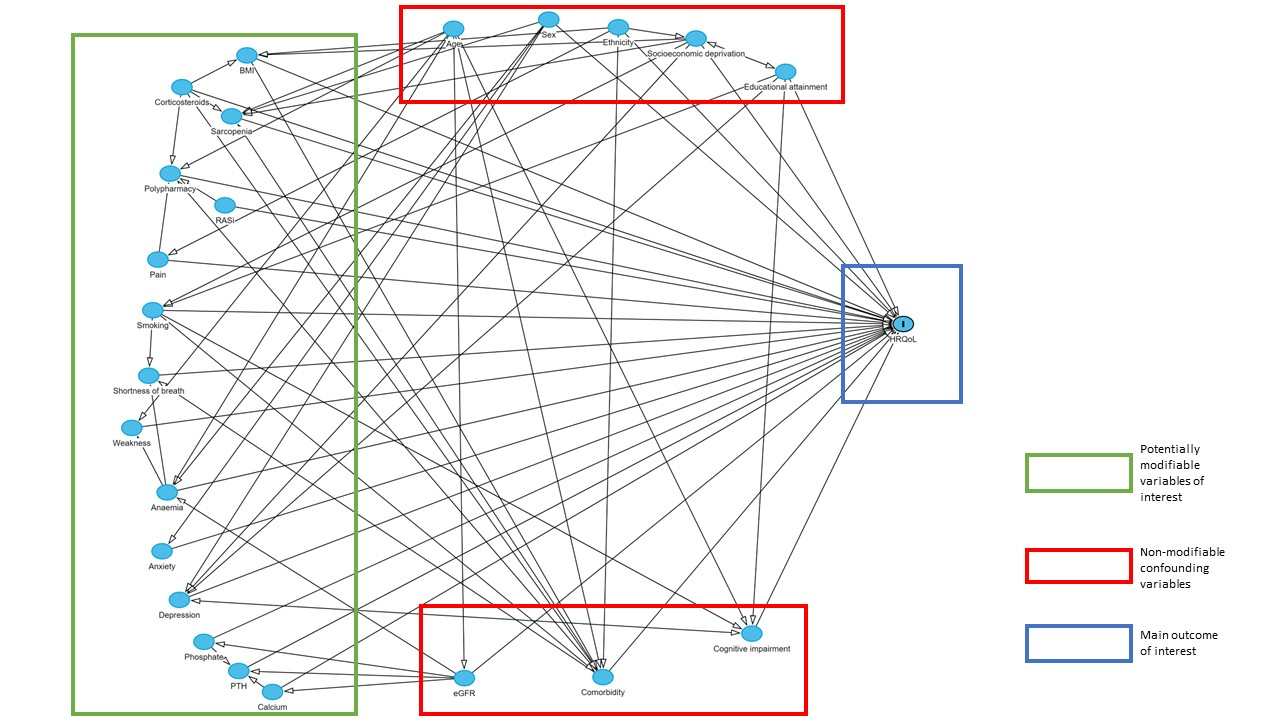

Supplement: sfae010_Supplemental_File [file sfae010_supplemental_file.docx]
